# Risk factors for breast cancer brain metastases: a systematic review

**DOI:** 10.18632/oncotarget.27453

**Published:** 2020-02-11

**Authors:** Lola Koniali, Andreas Hadjisavvas, Anastasia Constantinidou, Kyproula Christodoulou, Yiolanda Christou, Christiana Demetriou, Andreas S. Panayides, Constantinos Pitris, Constantinos S. Pattichis, Eleni Zamba-Papanicolaou, Kyriacos Kyriacou

**Affiliations:** ^1^Department of Electron Microscopy/Molecular Pathology, The Cyprus Institute of Neurology and Genetics, Nicosia, Cyprus; ^2^The Cyprus School of Molecular Medicine, The Cyprus Institute of Neurology and Genetics, Nicosia, Cyprus; ^3^Medical School, University of Cyprus and the Bank of Cyprus Oncology Centre, Nicosia, Cyprus; ^4^Neurogenetics Department, The Cyprus Institute of Neurology and Genetics, Nicosia, Cyprus; ^5^Neurology Clinic D, The Cyprus Institute of Neurology and Genetics, Nicosia, Cyprus; ^6^Department of Primary Care and Population Health, University of Nicosia Medical School, Nicosia, Cyprus; ^7^Department of Electrical and Computer Engineering, School of Engineering, University of Cyprus, Nicosia, Cyprus; ^8^Department of Computer Science, University of Cyprus, Nicosia, Cyprus; ^*^These authors contributed equally to this work

**Keywords:** breast cancer, brain metastases, risk factors, biomarkers

## Abstract

Background: Brain metastasis (BM) is an increasingly common and devastating complication of breast cancer (BC).

Methods: A systematic literature search of EMBASE and MEDLINE was conducted to elucidate the current state of knowledge on known and novel prognostic factors associated with 1) the risk for BCBM and 2) the time to brain metastases (TTBM).

Results: A total of 96 studies involving institutional records from 28 countries were identified. Of these, 69 studies reported risk factors of BCBM, 46 factors associated with the TTBM and twenty studies examined variables for both outcomes. Young age, estrogen receptor negativity (ER-), overexpression of human epidermal factor (HER2+), and higher presenting stage, histological grade, tumor size, Ki67 labeling index and nodal involvement were consistently found to be independent risk factors of BCBM. Of these, triple-negative BC (TNBC) subtype, ER-, higher presenting histological grade, tumor size, and nodal involvement were also reported to associate with shorter TTBM. In contrast, young age, hormone receptor negative (HR-) status, higher presenting stage, nodal involvement and development of liver metastasis were the most important risk factors for BM in HER2-positive patients.

Conclusions: The study provides a comprehensive and individual evaluation of the risk factors that could support the design of screening tools and interventional trials for early detection of BCBM.

## INTRODUCTION

Breast cancer (BC) is the most frequently diagnosed cancer among women worldwide, accounting for over 1.67 million new cases annually [1]. It is also the second leading cause of brain metastases (BM), with the reported prevalence among BC patients ranging from 10–16% and reaching 20–36% when data from autopsy series are included [2, 3]. Patients typically present with progressive neurologic and motor deficits, with symptoms ranging from headache and nausea to personality change, seizures, paralysis and cognitive impairment [4]. Remarkably, BM is considered a late event in the progression of BC, occurring 2 to 3 years after initial diagnosis, and it is typically preceded by lung, liver and/or bone metastases [5, 6]. Yet, cases of direct BC to BM are not uncommon (~12% of BCBM cases), as the unique anatomy of the human brain is thought to be providing a sanctuary site for tumor cells [7]. Of note, a steady increase in the incidence of BCBM has been documented over the last decades, owing to wider utilization of magnetic resonance imaging (MRI), availability of newer systemic therapies and longer survival rates in the metastatic setting [8].

The current treatment paradigm for patients with BCBM consists of multimodal approaches that include whole brain radiation therapy (WBRT), surgery, and/ or stereotactic radiosurgery (SRS), depending on the patient’s performance status and the number, size and localization of brain lesions [9]. Due to the blood-brain barrier (BBB), systemic therapies (chemo, hormonal and targeted therapies) appear to be of limited clinical benefit. More recently, the application of SRS has proven particularly advantageous in cases with limited intracranial disease or surgically inaccessible tumors, increasing the median survival time of BCBM patients to more than 1 year [10–12].

Currently no systematic guidelines for routine screening of high risk asymptomatic patients exist; BCBM diagnosis is solely performed only after manifestation of symptoms [13, 14]. According to a large multi-institutional study which included BC patients from clinical trials, the 10-year incidence of BM was estimated at 5.2%, though a number of clinical and demographic features were reported to heighten the risk for BCBM [6]. An in depth understanding of the pathogenic drivers of BCBM could guide future research directions and aid the identification of patients who may benefit from medical interventions such as regular MRI screening, prophylactic cranial irradiation (PCI) or use of treatment regimens with better brain penetration. The present study therefore aims to provide a systematic review of prognostic studies pertaining to BCBM and highlight the current state of knowledge on known and novel prognostic factors associated with 1) the risk for BCBM and 2) the TTBM. Lastly, we discuss limitations of these studies and identify areas deserving further investigation.

## RESULTS

**Figure 1:**
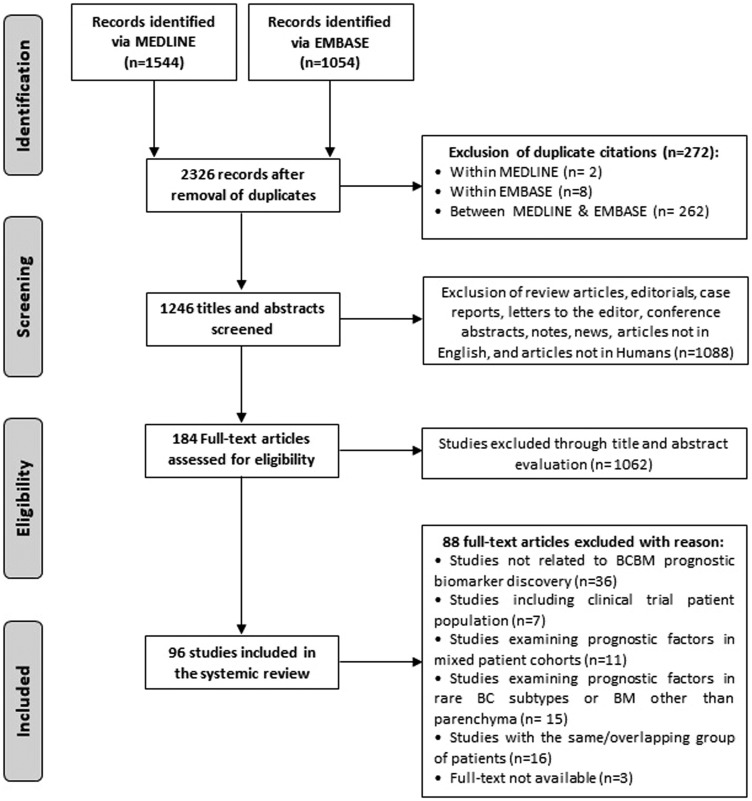
Flowchart of study selection.

Literature search yielded a total of 2598 publications including 1544 from MEDLINE and 1054 from EMBASE. After exclusion of duplicates and screening of titles and abstracts, a shortlist of 184 manuscripts were reviewed in full-text, and 96 of these were ultimately included in the review. Figure 1 provides a schematic representation of the pipeline used for the selection of studies. Eligible articles were published between 2002 and 2018, and involved institutional records and databases from 28 countries. Details of the studies included in the systematic review are presented in Supplementary Table 1.

In the light of the QUIPS ranking, eligible studies were deemed to have low to moderate overall risk of bias (Table 1). The complete results from the quality assessment of studies are provided in Supplementary Table 2. Study attrition was identified as the domain with the highest risk of bias as the majority was retrospective monocentric studies, where sample size was purely based on the availability of institutional records or databases. With respect to handling of cofounders, 26% of studies were deemed to have high risk of bias as no adjustment for confounding in multivariate analyses was performed. In studies where, multivariable statistical analysis was performed, adjustment parameters were typically variables exhibiting significance (*p* < 0.05) in univariate analysis (e. g. age, BC subtype, clinical stage at BC diagnosis); though cutoff *p*-value used across studies varied.

**Table 1:** Risk of bias in prognosis studies based on QUIPS scoring

Study domains and risk of bias	Studies (%)
**1. Study participation**	
Low risk	44.8
Moderate risk	45.8
High risk	9.4
**2. Study attrition** (Only applicable to cohort studies)	
Low risk	9.5
Moderate risk	23.8
High risk	66.7
**3. Prognostic factor definition and measurement**	
Low risk	76.0
Moderate risk	24.0
High risk	0.0
**4. Outcome definition and measurement**	
Low risk	81.3
Moderate risk	17.7
High risk	1.0
**5. Confounding measurement and handling**	
Low risk	59.4
Moderate risk	14.6
High risk	26.0
**6. Statistical analysis and reporting**	
Low risk	40.6
Moderate risk	51.0
High risk	8.3

### GROUP A - Prognostic factors associated with the risk for BCBM

In total, 69 studies were identified investigating predisposing factors for BCBM [10, 12, 15–82]. The majority of the included studies involved patients of all BC subtypes; thirteen reported risk factors for BM in HER2-positive patients [17, 20, 23–27, 44, 47, 61, 66, 80, 81] and three in TNBC patients [24, 39, 71]. Twelve studies compared clinicopathological characteristics of BCBM patients that developed BM as a first site of relapse, with those who first developed metastases elsewhere [10, 12, 21, 28, 30, 31, 39, 46, 54, 63, 66, 73]. In the majority of the studies potential risk factors for BCBM were assessed via univariate followed by multivariable analysis; variables were assessed via only univariate analysis in twenty-nine studies [10, 12, 15, 16, 19, 20, 22, 27, 30–32, 35–37, 41, 43, 45–48, 54, 56, 60, 65, 67, 68, 73, 77, 81] and via only multivariable analysis in five [25, 36, 50, 55, 74]. Tables 2 and 3 provide shortlists of the factors that were reported at least once to significantly and independently associate with the risk for BCBM, in studies with unselected BC patients and HER2-positive patients, respectively. Effect estimates measured by multivariate analysis are also displayed. More comprehensive lists of the comparison groups and the variables found to associate with BCBM via means of either univariate or multivariable statistical analyses in studies, with unselected BC patients and HER2-positive patients can be found in Supplementary Tables 3 and 4, respectively.

**Table 2:** Reported risk factors for BCBM by multivariate analysis in studies with unselected BC patient population

Variables	Studies measuring factor	Associated with increased risk for BCBM on univariate analysis	Significant result on multivariate analysis	
			Association with lower risk for BCBM	Association with higher risk for BCBM
**Age at primary BC/ MBC diagnosis**	**22** [10, 12, 16, 19, 21, 24, 28, 29, 33, 36, 38, 50, 52, 53, 55, 57, 59, 70, 74, 75, 78, 79]	**16** [12, 16, 19, 21, 24, 28, 29, 33, 38, 52, 53, 57, 70, 75, 78, 79]	**3** Continuous, AHR= 0.97, *p* = 0.024 [38]; >35 years, OR: 0.884 *p* = 0.02 [53]; Older age, HR = 0.943, *p* < 0.0001 [55]	**6** Young age, OR: 0.98 *p* < 0.001 [21]; <50 years HR: 2.0 *p* = 0.012 [24]; Young age, OR: 0.98 *p* < 0.001 [29]; ≤50 years, SHR: 1.97 *P* = 0.009 [33]; ≤35 years, HR: 2.09 *p* = 0.016 [57]; 41–60 years, OR:1.41 *p* = 0.02, 61–80 years, OR:1.40 *p* = 0.03 [74]
**ER status**	**15** [16, 18, 21, 33, 36, 37, 40, 51–53, 58, 59, 63, 75, 78]	**9** [16, 33, 37, 40, 51, 53, 58, 59, 75]	**2** ER+, HR 0.32, *p* = 0.002 [18]; ER+, HR:0.4 *p* = 0.025 [63]	**3** ER-, OR: 2.8 *p* < 0.001 [21]; ER-, HR:1.72 *p* = 0.029; [52] ER-, OR:5.027 *p* = 0.005 [53]
**HER2 overexpression**	**17** [18, 19, 21, 24, 35, 36, 38, 40, 51–53, 57, 59, 67, 73, 75, 78]	**9** [19, 35, 38, 52, 53, 67, 73, 75, 78]		**7** HR: 3.55, *p* = 0.006 [18]; HR: 3.4 *p* = 0.005 [24]; SHR: 2.58 *p* < 0.001 [33]; NR, *p* = 0.001 [51]; OR: 4.47 *p* < 0.05 [40]; OR: 7.039 *p* = 0.005 [53]; HR: 1.89 *p* = 0.049 [78]
**HER2 extracellular domain**	**1** [64]	**1** [64]		**1** Abnormal serum levels, HR: 4.25 *p* < 0.001 [64]
**HER2-positive (ER/PR±/HER2+) BC subtype**	**7** [24, 29, 41, 50, 51, 67, 70]	**5** [29, 41, 51, 67, 70]		**3** OR: 2.7 *p* < 0.001 [29]; HR: 3.4 *p* = 0.005 [24]; sHR: 3.41 *p* < 0.01 [70]
**ER-/PR-/HER2+ BC subtype**	**10** [12, 33, 55, 57, 63, 72–74, 78, 79]	**4** [33, 57, 73, 78]		**4** HR: 2.53 *p* < 0.001 [57]; OR: 2.09 *p* < 0.001 [74]; OR: 1.916 *p* < 0.001 [79]; HR-/HER2+, HR: 6.799, *p* < 0.0001 [55]
**TNBC (ER-/PR-/HER2-) BC subtype**	**21** [12, 24, 29–31, 36, 41, 42, 46, 50, 52, 54, 55, 57, 59, 63, 67, 70, 72, 74, 79]	**13** [29, 31, 36, 41, 42, 46, 52, 54, 57, 59, 67, 70, 72]		**10** HR: 4.2 *p* < 0.0001 [24]; OR: 1.4 *p* < 0.04 [29]; HR: 1.8 *p* < 0.003 [42]; HR: 5.5 *p* = 0.013 [50]; HR = 3.179, *p* < 0.001 [55]; HR: 4.42 *p* < 0.001 [57]; sHR: 2.08 *p* = 0.03 [70]; SHR: 2.10 *p* = 0.014 [72]; OR: 2.19 *p* < 0.001 [74]; OR: 1.749 *p* < 0.001 [79]
**HR+/HER2+ BC subtype**	**5** [24, 55, 72, 74, 79]	**2** [24, 72]		**3** HR+/HER2+, HR: 2.514, *p* < 0.001 [55]; SHR: 1.70, *p* < 0.037 [72]; OR: 1.41 *p* = 0.001 [74]
**HR+/HER2- Grade 3 BC subtype**	**1** [38]	**1** [38]	**1** AHR=0.18, *p* = 0.003 [38]	
**ER/PR+/HER2-/Ki67 high BC subtype**	**1** [63]			**1** HR:4.7 *p* = 0.0031 [63]
**Unknown vs. HR+/HER2-**	**1** [74]			**1** OR: 1.74 *p* < 0.001 [74]
**Ki67 labeling index**	**6** [36, 50–52, 69, 75]	**4** [36, 51, 69, 75]		**2** ≥30%, HR: 3.9 *p* = 0.026 [50]; ≥14%, HR: 2.76 *p* < 0.001 [52]
**Tumor histological type**	**9** [12, 16, 21, 28, 29, 33, 40, 52, 53, 70, 72, 78, 79]	**8** [21, 28, 29, 33, 40, 52, 70, 79]		**2** IDC, OR: 2.5 *p* = 0.02 [21]; lobular or mixed lobular + ductal), *p* = 0.033 [28]
**Tumor histological grade**	**19** [12, 16, 19, 28, 29, 33, 36, 38, 40, 50–53, 57, 59, 70, 72, 78, 79]	**12** [19, 28, 29, 36, 38, 51, 52, 57, 59, 70, 78, 79]		**5** High, *p* = 0.034 [28]; Grade 3, OR: 1.54, *p* = 0.01 [29]; High, OR: 13.4 *p* = 0.003 [36]; Grade 3, OR: 6.83 *p* = 0.002 [59]; unknown, OR: 2.428 *p* = 0.003 [79]
**Tumor nuclear grade**	**1** [28]	**1** [28]		**1** High, *p* = 0.046 [28]
**Clinical stage**	**6** [12, 29, 50, 55, 57, 78]	**3** [12, 57, 78]		**2** Stage III, HR: 4.836, *p* < 0.0001 [55]; Stage IV, HR: 67.07 *p* < 0.001 [57]
**Tumor size (T stage)**	**14** [10, 12, 18, 21, 24, 29, 33, 38, 40, 50, 53, 59, 70, 75, 78, 79]	**9** [10, 12, 18, 29, 33, 38, 53, 59, 75]		**5** >2cm, HR: 2.76, *p* = 0.013 [18]; 2.1–5cm, OR: 1.5, *p* = 0.01 [21]; T3/4, HR: 1.9 *p* = 0.02 [24]; >2cm, HR: 3.60 *p* = 0.003 [78]; 2, OR: 1.513 *p* = 0.045, 3, OR: 2.167 *p* = 0.001, 4, OR: 3.045 *p* < 0.001, Unknown, OR: 3.888 *p* < 0.001 [79]
**Lymph node status** **(N- Stage)**	**14** [12, 18, 19, 21, 24, 29, 33, 36, 38, 40, 53, 59, 75, 78, 79]	**8** [18, 19, 29, 36, 38, 53, 59, 78]		**5** Positive, HR: 2.4 *p* = 0.028 [24]; Positive, OR: 6.7 *p* = 0.042 [36]; ≥4, OR:8.390 *p* < 0.001 [53]; Positive, HR: 4.03 *p* < 0.001 [78]; 3, OR: 1.564 *p* = 0.017 [79]
**Metastatic status**	**4** [33, 38, 53, 70]	**3** [33, 38, 53]		**2** Primary metastatic status M0, SHR:2.64 *p* = 0.007 [33]; Extracranial distant metastases, AHR=28.46, *p* < 0.001 [38]
**Number of nonbrain metastatic sites**	**6** [29, 33, 55, 70, 74, 79]	**3** [29, 33, 70]		**5** >1 OR: 1.76, *p* < 0.001 [29]; ≥3, HR = 2.712, *p* < 0.0001 [55]; <1, sHR: 1.77 *p* = 0.04 [70]; 2, OR: 1.65 *p* < 0.001, 3, OR: 3.40 *p* < 0.001, unknown, OR:3.90 *p* < 0.001 [74]; 1, OR: 35.551 *p* < 0.001; 2, OR: 71.158 *p* < 0.001; 3, OR: 150.858 *p* < 0.001 [79]
**Time to distant relapse**	**5** [29, 53, 70, 72, 78]	**3** [29, 53, 72]		**2** Delay (/months), OR: 0.99, *p* = 0.02 [29]; <24 months, OR: 2.972 *p* = 0.01 [53]
**Bone metastases**	**1** [40]			**1** Absence, *p* = 0.035 [40]
**Vascular invasion**	4 [29, 38, 40, 72]	**1** [40]		**1** Peritumoral vascular emboli, SHR: 1.83 *p* = 0.005 [72]
**Rad51 cytoplasmic expression**	**1** [52]	**1** [52]		**1** Intermediate/high, HR: 1.87 *p* = 0.014 [52]
**Matrix metalloproteinase**	**1** [64]	**1** [64]		**1** Abnormal serum levels, HR: 3.51 *p* = 0.005 [64]
**p16 expression on metastatic lymph nodes**	**1** [69]	**1** [69]		**1** High score *p* = 0.01 [69]
***CRYAB* expression **	**1** [63]			**1** HR:1.2 *p* = 0.021 [63]
**3q gene signature**	**1** [76]			**1** HR: 1.61 *p* = 0.001 [76]
**GRP94 Status**	**2** [35, 62]			**1** Strong positive vs. negative [35]
**FN14 Status**	**2** [35, 62]			**1** Strong positive vs. negative [35]
**CTC status**	**1** [34]			**1** Undetectable CTC status OR: 6.17 *p* = 0.001 [34]
**SNPs**	**1** [72]			**1** AKT1 – RS3803304, SHR: 2.72 *p* = 0.008, AKT2 – RS3730050, SHR: 2.06 *p* = 0.041, PDK1 – RS11686903 SHR: 2.38 *p* = 0.001, PI3KR1 – RS706716, SHR: 2.42 *p* = 0.025 [72]

HR – Hazard Ratio; OR – Odds Ratio; NS – Non Significant; NR – Not Reported; SHR – Subhazard ratio; IDC – Infiltrating ductal carcinoma; CTC – Circulating tumor cells; AHR – Adjusted Hazard Ratio; sHR – subdistribution hazard ratio; SNPs – single-nucleotide polymorphism.

**Table 3:** Reported risk factors for BCBM by multivariate analysis in studies with HER2-positive BC patient population

Risk factor	Studies measuring factor	Associated with increased risk for BCBM on univariate analysis	Associated with increased risk for BCBM on multivariate analysis
**Age at primary BC/ MBC diagnosis**	**10** [17, 20, 23–26, 44, 61, 80, 81]	**2** [26, 80]	**5** NR, *p* < 0.05 [17]; <50 years, HR: 2.7, *p* = 0.0048 [25]; ≤50 years, HR: 1.92 *p* = 0.04 [26]; Young age OR: 1.66 *p* = 0.014 [44]; ≤40 years, *p* = 0.045 [80]
**Hormone receptor status**	**7** [20, 26, 27, 44, 61, 66, 80]	**3** [20, 44, 66]	**2** Negative, OR: 1.75 *p* = 0.033 [44]; Negative, RR: 3.41 *p* = 0.01 [66]
**Primary tumor size (T stage)**	**4** [24, 26, 66, 80]	**3** [24, 66, 80]	**1** >2 cm, HR:4.94 *p* = 0.0036 [80]
**Lymph node status (N- Stage)**	**4** [17, 24, 26, 80]	**1** [80]	**2** NR, *p* < 0.01 [17]; N1–3, *p* = 0.0045 [80]
**Number of nonbrain metastases**	**2** [26, 61]	**2** [26, 61]	**1** ≥2, OR: 8.30 *p* < 0.001 [61]
**Clinical stage/type**	**4** [26, 44, 61, 66]	**2** [26, 66]	**3** Recurrence, HR: 2.51, *p* = 0.02 [26]; Stage III, OR 2.05 *p* = 0.020 [44]; Stage III, RR: 9.39 *p* = 0.0032 [66]
**Time to distant relapse**	**4** [23, 26, 61, 80]	**2** [23, 80]	**1** ≤2 years, HR: 1.62 *p* = 0.022 [23]
**Liver metastases**	**4** [17, 26, 61, 102]	**2** [26, 61]	**2** NR, *p* < 0.01 [17]; [26] HR: 2.1, *p* = 0.04
**Lung metastases**	**3** [17, 26, 80]	**1** [80]	**1** lung (not *de novo*), HR: 6.97 *p* < 0.0001 [80]
**Adjuvant trastuzumab use**	**7** [17, 23, 24, 44, 47, 61]	**2** [47, 61]	**3** OR:1.61 *p* = 0.025 [80]; Late (≥6 months after BC diagnosis), HR: 2.65, *p* = 0.043, No, HR:3.79, *p* = 0.0042 [44]; ≥2 line OR: 3.43 *p* = 0.003 [61]

HR – Hazard Ratio; OR – Odds Ratio; RR – Relative risk; NR – Not Reported.

#### Age

The effect of age on the risk for BCBM progression was examined in 22 studies with unselected BC patient populations; six studies compared the mean/median age of patients with and without BM [10, 21, 36, 52, 59, 70], ten studies assessed age as a di or trichotomous variable [16, 19, 24, 33, 38, 50, 53, 57, 75, 78], and six assessed age as a continuous variable [29, 55, 63, 71, 72, 79]. Despite the marked heterogeneity in metrics and cutoff points used, young age at primary or metastatic breast cancer (MBC) diagnosis was constantly associated with BCBM, with fourteen studies reporting a statistically significant univariate association [16, 19, 21, 24, 29, 33, 38, 52, 53, 57, 70, 75, 78, 79] and eight studies a statistically significant multivariable association with effect estimates ranging between 0.97 – 2.0 [21, 24, 29, 33, 38, 53, 55, 57]. Importantly Aversa et al. calculated a 6% decrease in the risk for BCBM for every additional year of age at BC diagnosis [55].

By contrast, in studies comparing clinicopathological characteristics of BCBM patients that developed direct BM with those that first developed metastases elsewhere [10, 12, 21, 28], a significant higher incidence of direct BM was observed among older age patients (≥40 and ≥55 years) in two cohort studies, assessing age as a dichotomous variable [12, 28]. However, the trend did not reach statistical significance in two case-control studies comparing the mean/median age of patients between the two groups [10, 21]. Interestingly, in a large retrospective study examining prognostic factors in patients with synchronous BM and BC, older age (>40) was found to be a significant predictor of BCBM, in multivariable analysis (OR: 1.41, *p* = 0.02 for 41–60 years and OR: 1.40, *p* = 0.03 for 61–80 years) [74].

Unlike the general BC population, the effect of age on the risk for BCBM in TNBC and HER2-positive patients was not as prominent. Five studies reported a statistically significant multivariable association between young age and risk for BCBM with effect estimates ranging between 1.66 – 2.7 [17, 25, 26, 44, 80], whilst no significant association was found in the rest of the studies considering solely HER2-positive patients, likely reflecting the small sample size used [20, 23–25, 61, 81]. Similarly, in the subset of studies involving TNBC patients, age was not found to impact BCBM [24, 39, 71].

#### Menopausal status

Of the eight studies that examined the effect of menopausal status on the risk for BCBM [21, 28, 29, 38, 40, 50, 52, 53], five reported a significant higher incidence of BM among premenopausal than peri- or postmenopausal women [21, 28, 29, 38, 52]. Multivariable analysis adjusting for age in three studies revealed no significant association [21, 29, 50].

Of note, menopausal status was not found to associate with direct BCBM [21] or BM progression in HER2-positive [23, 61, 66, 83] and TNBC patients [39].

#### ER, PR and HER2 statuses and IHC BC subtypes

Negative ER status was found to significantly associate with higher risk for BCBM in 13 [16, 18, 21, 33, 37, 40, 51–53, 58, 59, 63, 75] of the 15 studies examined [16, 18, 21, 33, 36, 37, 40, 51–53, 58, 59, 63, 75, 78], with five reporting independent prognostic significance of ER-status following multivariable analyses (effect estimate range: 1.72 – 5.027 for ER-) [18, 21, 52, 53, 63].

Unlike ER, negative PR status was found to significantly associate with increased risk for BCBM only on univariate analysis [18, 21, 51–53, 59, 75].

In studies considering solely HER2-positive patients, multivariate analysis revealed HR-negativity to associate with a statistically significant higher risk of brain as the first relapse site (effect estimate range: 1.75 – 3.41) [44, 66]; however the trend did not reach statistical significance when ER/PR statuses were compared between HER2-positive BM and non-BM cases overall [23, 26, 27, 44, 80, 81].

HER2 protein expression in primary BC tumors was also found to associate with higher risk for BCBM [19, 35, 38, 52, 53, 67, 73, 75, 78] with seven studies reporting statistically significant multivariable associations (effect estimate range: 1.89 – 7.039) [18, 24, 40, 51, 53, 57, 78]. Of note, according to the study of Darlix and colleagues, elevated serum HER2 extracellular domain was found to accurately discriminate between BC patients with and without subsequent BM (HR: 4.25, *p* < 0.001) [64].

The prognostic value of immunohistochemistry (IHC) defined BC subtypes in predicting BCBM progression was examined in 28 studies [12, 24, 29–31, 33, 36, 38, 41–43, 46, 49–52, 54, 55, 57, 59, 63, 67, 70, 72–74, 78, 79]. Irrespective of the heterogeneity in the definition of BC subtypes and IHC cut-offs used for calling ER/PR positivity across studies (Allred scoring system or >1%, >5% and 10% staining as cut-offs for ER/PR positivity), the TNBC (effect estimate range: 1.4 – 5.5) [24, 29, 31, 36, 41, 42, 46, 50, 52, 54, 55, 57, 59, 67, 70, 72, 74, 79] and/or HER2-positive subtypes (HR-/HER2+ or HR±/HER2-) were constantly found to associate with a significantly higher cumulative incidence of BM (effect estimate range: 1.916 – 6.799) [12, 24, 29, 43, 49, 50, 55, 67, 70, 72–74, 78, 79]. Of note, comparison of the two HR-positive groups revealed HR/HER2 co-positivity to associate with significant higher incidences of BCBM progression over HR+/HER2- tumors [24, 43, 55, 72, 74, 79] with three studies reporting statistically significant multivariate association (HR: 2.514, *p* < 0.001; SHR: 1.70, *p* < 0.037; OR: 1.41, *p* = 0.001, respectively) [55, 72, 74].

Regarding studies assessing the effect of BC subtypes on the risk of BM as the first site of relapse, results were contradicting with four studies indicating a significantly higher propensity of HER2-positive and/or TNBC tumors to metastasize to BM first [31, 41, 54, 67] and two studies reporting no significant association [30, 84].

#### Other markers

Additional primary tumor markers found to associate with BCBM progression by more than one study included Ki67, EGFR, FOXC1 and CK5/6. Although different IHC staining cut-offs were used across studies, high expression of cellular proliferation biomarker Ki-67 was consistently found to significantly associate with BCBM progression both on univariate [36, 51, 52, 75] and multivariate analysis (effect estimate range: 2.76 – 3.9) [50, 52]. Similarly, Furet et al. reported a significantly higher percentage of Ki67-expressing cells in the lymph node biopsies of HER2-positive and TNBC patients who subsequently developed BM [69]. Additionally, EGFR [15, 19, 21, 36, 52], and CK5/6 [19, 37, 52], which are well known markers of the basal BC subtype, were reported by a series of studies to associate with BCBM; whilst, univariate analysis in two independent studies revealed elevated FOXC1 gene expression to associate with BCBM [32, 60]. Consistent with these studies, Luck et al. reported an increased risk of BM among BC patients with basal phenotype (defined by expression of CK5/6 and/or CK14 in 10% or more of the tumor cells) [22]. More recently, concomitant expression of GRP94, FN14, and inhibin in primary tumors of BC patients was reported to be predictive of subsequent BCBM [35, 75].

#### Primary tumor histology

With regards to primary tumor histology, conflicting results were observed across studies [12, 16, 21, 28, 29, 33, 40, 52, 53, 70, 72, 78, 79]. Six studies supported a link between invasive ductal carcinoma (IDC) and BCBM [21, 29, 33, 40, 70, 79]; and two studies reported a significant higher incidence of BCBM in patients with lobular carcinoma versus IDC [28, 52]. Additionally, primary tumor histology was not found to associate with direct BM progression [21] or the development of BM in HER2-patients [23, 61].

#### Primary tumor grade

High primary tumor histological grade (G3 vs. G1–2) was recurrently associated with BCBM progression, with twelve [19, 28, 29, 36, 38, 51, 52, 57, 59, 70, 78, 79] of the 19 studies examined [12, 16, 19, 28, 29, 33, 36, 38, 40, 50–53, 57, 59, 70, 72, 78, 79], reporting a statistically significant univariate association. On multivariate analysis, a significant positive association between Grade 3 and BCBM progression was observed in five studies (effect estimate range 1.54 – 13.4) [28, 29, 36, 59, 79]. Intriguingly, risk for BCBM was found to be independent of primary tumor grade in the subset of studies, involving solely HER2-positive [20, 23, 25, 26, 61, 66, 80] or TNBC patients [24, 39].

#### T, N and M statuses and tumor stage

The effect of TNM staging, (according to the American Joint committee on Cancer criteria which incorporates Tumor size, Nodal status and Metastatic involvement) was examined in six studies [12, 29, 50, 55, 57, 78]; however only two reported a significant association between stage III/IV disease and BCBM progression in multivariate analysis (effect estimate range: 4.836 – 67.07) [55, 57]. Interestingly, multivariable analysis in three of the four studies involving solely HER2-positive patients [26, 44, 66], revealed a significant positive association between stage III/recurrent disease and the risk for BCBM (HR:2.51, *p* = 0.02; OR:2.05, *p* = 0.020; RR:9.39, *p* = 0.0032, respectively). Of note, TNBC patients presenting with advanced stage disease were found to associate with significantly higher incidence of brain as a first site of relapse in a single-institutional study [39].

Examined separately, all three factors (Tumor size, Nodal status and Metastatic involvement) were found to predict BCBM. Irrespective of metrics and cut-off points used, patients presenting with primary BC tumors bigger than 2 cm (≥T2) were found to significantly associate with higher risks for BM in all studies examined [12, 18, 21, 24, 29, 38, 53, 59, 75, 79], but four [33, 40, 50, 70]; whilst five studies reported independent prognostic significance following multivariable analysis (effect estimate range 1.5–3.6) [18, 21, 24, 78, 79]. The effect of tumor size on the risk for BCBM was less prominent in studies comparing patients with T1–2 versus T3–4 status at primary diagnosis [33, 50].

Similarly, in studies considering only HER2-positive patients [24, 26, 66, 80], increased tumor size was found to significantly associate with BM progression on univariate analysis in three studies [24, 66, 80]. Only one study involving HER2-positive patients, reported significant multivariate association between tumor size and BCBM (>2 cm, HR:4.94) [80]. Separate analyses in a study considering solely TNBC [71] revealed increased tumor size to significantly associate with BM progression on multivariate analysis (HR:4.632, *p* = 0.0071).

The prognostic relevance of nodal involvement on the risk for BCBM was largely dependent on the study population. Risk for BM among BC patients was significantly associated with positive nodal status at primary diagnosis [12, 18, 19, 24, 29, 36, 38, 59, 78, 79], with five studies [24, 36, 53, 78, 79] reporting statistical significant multivariable association (HR:2.4, *p* = 0.028; OR:6.7, *p* = 0.042; OR:8.390, *p* < 0.001; HR:4.03, *p* < 0.001; OR:1.564, *p* = 0.017, respectively). However, this association did not reach statistical significance in the subset of studies where a case-control analysis between non-brain MBC and BCBM patients was performed [21, 33, 40, 75]. Of note, Demircioglu et al. also determined significance of lymph node ratio (percentage of lymph nodes with metastases over the number removed) in predicting BCBM [48]. Similarly, in studies involving HER2-positive BC patients [17, 24, 26, 80, 81], positive nodal status was found to significantly and independently associate with BCBM in only two of the studies examined [17, 80].

Metastatic disease itself [33, 38, 53], and increasing number of metastatic sites were also found to be independent adverse prognostic factors for BCBM progression, in both the general BC population (effect estimates range 2.64–28.46 and 1.65–150.858, respectively) [29, 33, 38, 55, 70, 74, 79] and in patients with HER2-positive disease [26, 61]. Further to these findings, a short time interval between initial BC diagnosis and metastatic dissemination was also reported to be an independent predictor of BCBM progression, in two studies involving unselected BC patients (effect estimate range: 0.99 – 2.972) [29, 53] and in one study considering solely HER-2 positive patients [23].

#### Metastatic sites

The prognostic significance of different metastatic sites was also examined across studies, with varying results. Two independent studies reported a significant positive association between vascular invasion and BCBM progression in univariate analysis [40, 72]. Similarly, lymphovascular invasion was found to associate with significant higher cumulative incidence of BM in a single study involving TNBC patients [39]; but not in a separate one involving early stage BC patients [50].

Despite studies indicating no effect of first site of metastasis on the risk for BCBM [53, 72, 78], a number of studies found significant higher risk for BCBM in patients presenting with visceral [29], lung [33, 51], and lymph node [33] as first sites of metastasis. Importantly, in the majority of the studies involving HER2-positive patients, liver metastasis was found to significantly associate with BCBM progression, both following univariate [26, 61] and multivariable analysis [17, 26]. In only one study, lung as first site of relapse was found to be an independent risk factor for BM progression in HER2-positive patients (HR: 6.97, *p* < 0.0001) [80]. Bone metastasis was also reported by a single study to associate with the development of BCBM in HER2-positive patients but only on univariate analysis [26].

#### Treatment-related factors

The effect of various chemotherapy regimens and in particular trastuzumab on the risk for BCBM was assessed in a number of studies with variable results. Omission of trastuzumab was not found to associate with the risk for BCBM in all studies examined [17, 23, 24, 52] but two [44, 47]. The exceptions were the study by Yap et al. where trastuzumab use was found to associate with increased risk for BM as first site of metastasis [47] and the study of Vaz-Luis et al. where trastuzumab use was found to independently associate with higher incidence of BM over other sites of metastasis (OR: 1.61 [*p* = 0.025]) [44]. Similarly, late start (>6 months) or omission of trastuzumab after surgery, were also reported to independently associate with increased risk for BM in a single study (HR:2.65, *p* = 0.025 and HR:3.79, *p* = 0.0042, respectively) [80]. Additionally, in a single case-control study conducted in Turkey, administration of 2 or more lines of trastuzumab, were found to be an independent risk factor associated with increased risk for BM (HR:3.43, *p* = 0.003) [61].

### GROUP B - Prognostic factors associated with the TTBM

In total, 46 studies investigating the effect of variables on TTBM were identified. In thirty-six studies, the TTBM interval was measured from initial BC diagnosis [10–12, 24, 32, 39, 46, 47, 54, 63, 68, 76, 78, 85–104]; in six studies from presentation of first distant/extracranial metastasis [55, 56, 61, 70, 84, 105] and in one study from the initiation of BC treatment [83]. Five studies reported prognostic factors influencing TTBM measured from both time-points [28, 30, 38, 58, 106]. Four studies reported on factors associated with the TTBM in solely HER2-positive patients [47, 61, 83, 103, 104] and one in TNBC [39]. The majority of the eligible studies were limited to reporting univariate association between BC subtypes or a single candidate factor with TTBM. Only fourteen studies provided multivariable TTBM estimates for variables [11, 28, 39, 58, 63, 70, 76, 83, 84, 90, 94, 96, 102, 104].

#### Age and menopausal status

With the exception of a single study reporting shorter TTBM as first site of relapse in patients >35years and stage III at primary diagnosis [96], age was considered but not found to significantly associate with early onset of BCBM progression [11, 28, 39, 58, 70, 78, 83, 84, 90, 94, 96, 106]. Additionally, controversial results were reported across four studies accessing the effect of menopausal status on the TTBM [15, 58, 90, 106].

#### ER, PR and HER2 statuses and IHC BC subtypes

The impact of IHC-defined BC subtypes on TTBM was examined via multivariable analysis in only five studies [58, 70, 84, 90, 94], of which three [70, 84, 90] reported significant multivariable associations (HR:2.0, *p* = 0.004 for HR-/HER2+, HR:1.51, *p* = 0.0002 for non-luminal BC subtypes, and sHR: 2.08, *p* = 0.03 for HR-/HER2-, respectively). Univariate analysis in the rest of the studies revealed significant differences in TTBM among BC subtypes; though variability in the classification of BC subtypes and in the cut-off points used for calling ER/PR positivity was observed. With the exception of one study where only a small proportion of the patients received hormonotherapy or HER2 targeted therapies [106], studies that stratified patients into four BC subtypes (HR+/HER2-, HR+/HER2+, HR-/HER2+ and HR-/HER2-) and measured TTBM interval from primary BC [28, 38, 54, 86, 90, 92, 97, 100, 104, 105] or MBC diagnosis [28, 38, 55], the non-luminal subtypes (HR-/HER2- and HR-/HER2+) were found to associate with significantly shorter TTBM, compared to the luminal ones (HR+/HER2-, HR+/HER2+). Comparison of the two HR-positive groups revealed HR/HER2 co-positivity to constantly associate with shorter TTBM [84, 102]. Similar results were observed across seven studies [24, 30, 70, 84, 91, 97, 98] stratifying patients into three BC subtypes (HR+/HER2-, HR±/HER2+, and HR-/HER2-), with the TNBC tumors demonstrating the shortest TTBM (20–25.5 months from primary diagnosis and 9–14 months from MBC) and HR+/HER2- the longest (42–63.5 months from primary diagnosis and 20.6–34 months from MBC). Interestingly, in two studies stratifying BCBM patients into HR+, HR-/HER2+ and HR-/HER2-, no significant difference in the median time interval between BC and BM diagnosis [94], or first BM treatment [89] were observed, across BC subtypes. Similarly, of the seven studies that compared TTBM between TNBC and non-TNBC cases [10, 12, 87, 95, 99, 105], 4 reported no significant difference between the two groups [10, 46, 87, 95]. Importantly, according to the study of Fokas and colleagues, the median TTBM for TNBC patients significantly differed from that of the other BC subtypes, only during the first 16 months from BC diagnosis [87].

Examined alone, ER status, but not PR or HER2 statuses, seemed to influence the TTBM [10, 11, 28, 58, 88, 90, 96, 102, 104, 106]. In total, seven studies investigated the association between ER tumor status and TTBM; two reported significant univariate associations [10, 28] and three studies [58, 102, 104], including one involving solely HER2-positive patients [102], significant multivariate associations (effect estimate range: 1.36 – 5.1). By contrast, of the four studies that assessed the effect of PR-status on the TTBM [11, 28, 58, 104], one reported independent prognostic significance of PR-status on multivariate analysis (HR:1.31, *p* = 0.021 for PR-negative tumor) [104] and one reported a positive univariate association [58]. Additionally, with the exception of a single study [96], HR-positivity (defined as ER and/or PR positive) was found to significantly associate with longer TTBM from MBC dissemination [106] or primary disease diagnosis [88, 104].

Similarly, independent of HR levels, HER2-status was not found to associate with TTBM in all studies examined [10–12, 28, 58, 87, 88, 95, 96, 102, 106] but two [101, 104] that reported borderline statistically significant univariate associations and two [70, 90] more that reported independent prognostic significance of HER2-status on multivariate analysis (HR: 2.0, *p* = 0.004 and sHR:3.41, *p* < 0.0001, respectively). Remarkably, in one study examining correlation between quantitative HER2 protein levels (via the HERmark assay) and TTBM, Duchnowska and colleagues observed increased levels of HER2 protein to associate with shorter TTBM [83]. The same group also reported on the prevalence and prognostic significance of HER2 protein and its truncated, constitutively active form (p95HER2) in 75 pairs of BC/BM FFPE samples. p95HER2 was detected in over 33% of primary BC samples and was found to trend with shorter TTBM [107]. Of note, quantitative levels of HER2 protein via the HERmark assay in this patient cohort, did not confirm earlier findings.

#### Primary tumor grade

High primary tumor grade (G3 vs. G1–2) was also found to be an unfavorable prognostic factor for the TTBM. Three studies [28, 96, 104] reported significant multivariate associations (*p* ≤ 0.04; *p* = 0.04 and HR: 1.95, *p* = 0.023, respectively) and four studies [70, 78, 83, 90], including one involving HER2-positve patients [83], significant univariate associations.

#### T, N and M statuses and clinical stage

Not surprisingly, advanced stage disease (Stage ≥ III or stage IV) at primary BC diagnosis was predictive of shorter TTBM [11, 90, 92, 94, 96, 106]; with four studies [11, 94, 104], including one involving solely TNBC cases [39] reporting a statistically significant multivariable associations: Stage III/IV, HR: NR, *p* = 0.033/ 0.047; HR: 3.389/6.643, *p* = 0.016/0.025; HR: 3.96, *p* < 0.001; HR: 3.51, *p* = 0.001, respectively. The exceptions were three studies, one involving early stage/non metastatic patients [30], one measuring TTBM from time of first distant metastasis [84], and the other comparing localized to regional stage disease [58]. Examined alone, large tumor size at presentation (T2–3 vs. T1) was also found to associate with significantly shorter TTBM on univariate analysis in two studies [10, 78], though different cut-offs and reference groups were used across the rest of the studies [10, 28, 70]. Similarly, positive axillary lymph node status was reported to be an adverse prognostic marker of BM-free survival in two [10, 78] of the three studies examined [58].

Primary metastatic status (HR:3.76, *p* < 0.001) [104] and the number of non-brain metastatic sites [70, 104] were also found to associate with shorter TTBM. Likewise, with the exception of a single study that reported borderline statistical significance on multivariate analysis [70], metastases-free interval of less than 1 year according to the study of Saip et al. [28] and less than 2-years in two separate studies [84, 104] were significantly associated with shorter TTBM interval (HR:1.49, *p* = 0.019) [84]. Similarly, time to non-brain progression was also found to correlate with the TTBM in a study involving HER2-positive patients [83].

In line with these, presence of pulmonary metastases (HR:1.49, *p* = 0.019) [84] or vascular invasion [30], were independently reported to be adverse prognostic factors for TTBM. Presence of visceral metastases was also found to associate with shorter TTBM on univariate in one of the three studies that examined the association [94], and on multivariable analysis in a single study involving advanced HER2-positive patients (HR: 7.4, *p* < 0.001 and HR:6.1, *p* = 0.01 in cohort A and B, respectively) [102]. Additionally, Darlix et al. reported significantly shorter TTBM among patients with no bone, liver and lymph node metastases prior to BM.

#### Histological subtype

Of the five studies that examined association of primary tumor histology with TTBM [28, 70, 90, 94, 104], invasive lobular carcinoma (ILC) was found to be an independent adverse prognostic factor of TTBM by only one study (HR: 6.911, *p* = 0.005) [94]. On the contrary, in two independent studies ductal and ductal/ lobular histology tumors were found to associate with significantly shorter TTBM compared to other histological subgroups, on univariate analysis [70, 104].

#### Treatment related factors

Not surprisingly, omission of systemic palliative treatment (*p* ≤ 0.0001) [96] and omission of adjuvant hormonotherapy in HR-positive patients, were found to associate with decreased TTBM [84, 90]. Similarly, TTBM was also found to be significantly shorter in chemo-resistant BC patients (complete/partial vs. stabile/progression, *p* = 0.037) [28]. Additionally, the administration of taxane-anthracycline combination was reported to associate with shorter TTBM than anthracycline-based regimen [106]. Intriguingly, administration of trastuzumab in HER2-positive patients was found to associate with a significantly longer TTBM in three studies involving solely HER2-positive patients [47, 102, 103]. However, this result was not observed for HER2-positive patients in studies including unselected BC population [84, 90, 96, 104]. Of note, in one study trastuzumab administration was found to associate with significantly shorter TTBM following multivariate analysis; though patients who received trastuzumab were older and had extra-cerebral metastases more frequently [104].

### GROUP C - Potential genetic and molecular markers associating with BCBM

The literature search also identified sixteen studies that attempted to correlate proteins or gene expression profiling data with incidence of BC or TTBM; though further work is warranted to assess the clinical utility of these results [32, 34, 37, 56, 63–65, 68, 69, 72, 76, 77, 82, 85, 93, 98]. Primary tumor biomarkers reported to associate with BCBM progression included, p53 mutational status, fraction of cells in the S-phase of the cell cycle, DNA ploidy, and levels of Bcl-2 [21], p63 [36], KISS-1 [93], nuclear Rad51, cytoplasmic Rad51, cytoplasmic CXCR4, nuclear CXCR4 [52], HER3 [56], aB-crystallin (HR:1.2, *p* = 0.021) [63] and CDKN2A/p16 protein [69]. Of note, in the study of Sosinska-Mielcarek et al., a profile of three-protein markers (ER-, Ki-67 ≥14% and cytoplasmic Rad51-positive) was associated with particularly high risk of BCBM (HR:4.43, *p* < 0.001) [52].

Not surprisingly, a number of proteins involved in tumor cell dissemination and BBB invasion were also found to associate with BCBM progression, including cathepsin-S [85], S100A4 [68], RANKL, RANK, Src [65], HYAL1, HAS2 [77] and prominin-1 (CD133) [37]. Darlix and colleagues also reported on serum biomarkers, originally found to reflect central nervous system damage in neurological diseases (NSE and MMP-9), to also significantly associate with BCBM progression following multivariate analysis [64].

In line with the prognostic relevance of the human epidermal growth factor receptor family (EGFR, HER2, HER3) in BCBM progression, a number of studies also investigated the prognostic value of EGFRs downstream mediators in risk for BCBM (PTEN, PIK3CA, AKT, mTOR). One study identified single nucleotide polymorphisms in genes encoding for components of the PI3K-AKT-mTOR pathway (AKT1-rs3803304, AKT2-rs3730050, PDK1-rs11686903, PI3KR1-rs706716) to associate with BCBM progression both on univariate and multivariable analyses [72]. Additionally, Adamo and colleagues found PTEN loss to associate with shorter TTBM [98]; whilst Qian and colleagues via integrating data from 3 independent datasets (EMC344, GSE12276, GSE2603) showed a 3q 19-gene signature (including PIK3CA, FXR1 SOX2, DCUN1D1, TP63, EIF4G1, EVI1, THPO, TERC, ECT2, PRKCI, EPHB3, MASP1 and SST) to independently associate with increased risk and shorter TTBM (HR: 1.61, *p* = 0.001) and/or lung metastases [76].

The prognostic value of circulating tumor cells (CTCs) was also examined in two independent studies. In a single center study employing the CellSearch^™^ system for measuring CTCs, revealed negative CTC-status in whole peripheral blood of BC patients to positively associate with BM [34]. Whilst more recently, Boral and colleagues, using stem-like markers (CD44^+^/CD24^−^) for isolating CTCs found a unique CTC-gene signature associating with re-proliferative ability of stem cells to be predictive of BCBM [82].

### GROUP D - Models predicting risk for BCBM

In the past decade, a number of BCBM predictive models have been proposed on the basis of known and novel risk factors; though with variable results [29, 53, 71, 78, 102]. Table 4 provides details on the methodological aspects of these models. First, the nomogram by Graesslin et al., which was recently validated by an independent study [70], takes into consideration age, grade, ER/PR/HER2 statuses, duration of metastasis free survival and number of non-BM sites. Secondly, Xue and colleagues proposed a nomogram based on 4 clinicopathological factors including number of metastatic axillary lymph nodes, ER/HER2 status, age at diagnosis, metastasis free survival; however the prognostic value of this model was found to be only moderate. Similarly, Azim et al. proposed a predictive score (Brain Relapse Index) based on HER2 positivity, axillary nodal status and tumor size [78]. More recently, in an independent study employing two cohorts of advanced HER2-positive patients (discovery and validation), Duchnowska and colleagues identified a 13-gene expression signature via DASL array (CDK4, CCNC, PTK2, MYC, BARD1, RAD51, FANCG, PCNA, PRCC, TPR, CTTN, DSP, HDGF) that was predictive of BCBM in HER2-positive patients; though, the predictive role of a refined 3 gene-signature (RAD51, HDGF, TPR) by qRT-PCR was not confirmed in the validation cohort [102]. Similarly, Klimov et al. developed BCBM risk prediction model for TNBC cases [71].

**Table 4:** Details of the studies describing models for predicting BCBM

Source	Study Location	Methodology	Factors included in the prediction models	Comments
Graesslin et al. 2010 [29]	USA	Multivariable logistic regression was used; bootstrapping with 1000 resamples in the 2136-patient training dataset and independent external validation with 128-patients cohort	Age, Histological grade, status for ER/PR/HER2, delay between diagnosis and first metastasis (months) number of non-brain metastatic organs	Nomogram predicting the probability of BM at the time of MBC; AUC 0.68 (95% CI, 0.66 – 0.69); validation AUC 0.74 (95% CI, 0.70 – 0.79)
Xue et al. 2013 [53]	China	Multivariable logistic regression model using 206-patient dataset	Age, number of metastatic axillary lymph nodes, ER status, metastasis-free survival	Model with moderate ability and limited power in assessing the risk for BCBM; AUC 0.765 (95% CI, 0.688 – 0.842)
Duchnowska et al. 2015 [102]	Poland & Serbia	Built-in 10-fold cross-validation analysis for selection of best gene-expression signature & Multivariable Cox regression model were used; advanced HER2-positive BC patients divided into discovery (84) and validation (75) cohorts	13-gene expression signature (*CDK4, CCNC, PTK2, MYC, BARD1,* *RAD51, FANCG, PCNA, PRCC, TPR, CTTN, DSP,* *HDGF, ACTB, GAPDH, TFRC*) & 3-gene expression signature by qRT-PCR (*RAD51, HDGF, TPR*)	13-gene classifier (high vs low) distinguishing between patients with early (<3 years) vs. late BCBM in the discovery cohort; HR 8.5 (95% CI, 2.6–28) *p* < 0.001 3-gene classifier (high vs low) distinguishing between patients with early (<3 years) vs. late BCBM in the discovery cohort; HR 5.3(95% CI, 1.6–16.7) *p* 0.005 (not significant in the validation cohort)
Klimov et al. 2017 [71]	UK	Cox proportional hazards regression model was used; 322 stage I-IIIA TNBC	*Parp1* nuclear H score, BRCA2 cytoplasmic H, Nottingham Prognostic Index groups	Brain metastasis score; high-risk patients possessed >7 higher risk of BM; 36.362HR (95% CI, 4.276–309.228) *p* 0.005
Azim et al. 2018 [78]	Egypt	Cox proportional hazards regression model was used to identify independent factors in a cohort of 2193 and a score of 1 was assigned for every positive risk factor	HER2 positivity, axillary lymph node metastasis, tumor size	Brain Relapse Index predicts 5-year cumulative incidence of developing BM (19.2% for patients with BRI 2–3; 2.5% for patients with BRI 1)
Kim et al. 2018 [79]	USA, France	Multivariable logistic regression and the Hosmer and Lemeshow Goodness of Fit test were used; 206913 patients were randomly and evenly assigned into a training and validation set	Age, Grade, T stage, N stage, BC subtype, number of metastatic organs except brain	Nomogram predicting the probability of BM at the time of BC diagnosis; AUC 0.960 (95% CI, 0.951 – 0.970); validation AUC 0.955 (95% CI, 0.945 – 0.965)

^*^ER – estrogen receptor; PR – progesterone receptor; HER2 – human epidermal growth factor receptor 2; BM – brain metastases; MBC – metastatic breast cancer; AUC – area under the curve; CI – confidence interval; HR – Hazard ratio; BCBM – breast cancer brain metastases; BC – breast cancer; BRI – brain relapse index.

## DISCUSSION

To date, both in terms of patients’ survival and cost-effectiveness, little evidence exists to support the implementation of occult BCBM surveillance program. Results from two independent studies support no significant overall survival benefit in patients diagnosed with occult BM compared to the symptomatic ones [108, 109]. However, the treatment paradigm used in those studies differed from the one applied today, as none of the patients received surgery or SRS. With advances in BM treatment modalities, and recent evidence indicating limited number of BM lesions to associate with better survival of patients, it may be rational to design studies to evaluate the utility of early diagnosis of BM [10, 12, 54, 89, 90, 92, 106, 110, 111]. However, if a successful, cost-effective screening program is to be established for BCBM, an in depth understanding of the pathogenic drivers of BCBM and accurate identification of patients likely to benefit from such an intervention would be required. Via integrating data from 96 observational prognostic studies conducted across 28 countries, herein, we attempted to provide the largest and most comprehensive review on prognostic literature, pertaining to BCBM and elucidate the current state of knowledge on risk factors for BCBM.

A major strength of this review includes the systematic methodology that was used for the selection of studies, enabling the unbiased analysis of relevant studies and prognostic factors. Not surprisingly, our findings substantiate the prognostic relevance of a number of factors considered in BCBM risk prediction models including age, histological grade, ER and HER2 statuses, tumor size, number of metastatic sites, and metastasis-free survival. Significantly, whilst young age was determined to be an adverse prognostic factor for BCBM, incidences of direct BM were more common among older age patients. Additionally, an important outcome of this search was the identification of Ki67 labeling index and a large number of basal-phenotype markers that were reported to predict BCBM and could thus be considered in future models; though further work is warranted to assess their clinical utility. Moreover, the inclusion of studies investigating factors associated with the TTBM enabled the identification of factors associating with early onset of BCBM. Unfavorable prognostic factors for the TTBM on multivariate analysis included ER-negative tumor status, advance stage disease and BC subtype. However, variability in the IHC classification of BC subtypes seemed to greatly impact the effect. Thus, moving forward, use of consensus criteria would allow better data comparisons.

One of the most intriguing aspects of this review was the evidence from a subset of studies involving solely HER2-positive patients, suggesting potential variations in the risk factors for BM between BC subtypes. Unlike the unselected BC patient population where factors such as age, tumor grade and size were found to strongly predict BCBM, the association was not as prominent among the HER2-positive patients. Interestingly, development of liver metastasis was one of the most significant factors found to associate with BCBM. Whether, this is due to the propensity of HER2-positive tumors to metastasize to liver [112, 113], or potential overlap in the pathogenic drivers coordinating brain and liver metastases, remains to be determined. In support of this finding, in the study of Niwinska et al., investigating risk factors for occult BM in HER2-positive patients, visceral metastases (lung and/or liver) was determined to be the only significant factor predicting occult BM [109]. To this end, preventive strategies may improve survival of these patients. Indeed, preliminary results from a recent small clinical trial examining the utility of PCI in HER2-positive metastatic BC patients, hinted a potential role of PCI in reducing incidences of BM [114]. Thus, moving forward, considering BC subtypes as separate entities could assist in the identification of novel predictive biomarkers for BCBM as well as in the interpretation of the clinical significance of preventive and treatment strategies.

A number of limitations of our results deserve mentioning. Firstly, from a methodological point of view, loss of literature due to the search strategy should be considered. Secondly as with every systematic review that is based on previously published data; the lack of individual patient information is a drawback, increasing the chances of unforeseen variables and the risk of selection bias. Moreover, heterogeneity in the patient populations, study design, methods and reporting of prognostic factors across studies, made pooling of results impossible. Additionally, identified studies that reported association between variables and TTBM were typically of limited statistical power, as many of these studies were not designed to evaluate TTBM as the primary outcome. Moreover, the small number of identified studies considering solely TNBC patients has limited the ability to draw firm conclusions. A common limitation in the majority of these studies was the small sample size, which made use of multivariable analysis not possible. Selection bias due to the retrospective design of the majority of the eligible studies should also be considered. Of note, random sampling methods such as bootstrapping or use of validation cohorts which could increase power of results were generally underused in the majority of these studies.

In conclusion, BM is becoming increasingly common complication of BC, but the incidence, timing and outcome vary significantly among patients. Acquiring evidence on risk factors for BCBM progression is *per se* a challenging task given the BC subtypes’ distinct biology and treatment responses. Thus, an accurate prediction of BC patients at high risk of developing BM would entail the collaboration of a multidisciplinary team of expertise. In this context, a multilevel diagnostic tool integrating both clinical and laboratory variables would be necessary for optimal risk stratification and management of patients. To our knowledge, this study is the largest and most comprehensive review of literature on previously reported prognostic risk factors for BCBM. In the short term, the work presented here could provide a rational basis for the design of a prospective clinical trial assessing the utility of occult BM diagnosis and/or preventive medical interventions. In the long term, results could enable the refinement of prognostication tools for BCBM and assist clinicians during treatment-decision making process.

## MATERIALS AND METHODS

### Search strategy

A systematic literature review adhering to the PRISMA guidelines was conducted to identify studies investigating predictors of BCBM. Bibliographic databases, EMBASE and MEDLINE were searched from inception through January 5, 2019 using terms (“breast neoplasm” OR “breast cancer” OR “breast tumor”) AND (“brain neoplasm, malignant” OR “brain metastasis” OR “brain tumor” OR “brain cancer”) AND (“risk factors” OR “biomarkers” OR “prognostic markers” OR “biological marker”). Results were restricted to English language publications and human subject research. Book chapters, case reports, editorials, meeting abstracts, notes and review articles were excluded.

### Selection criteria

Study selection was performed by two independent investigators (LK and AH), and in cases of disagreement a third investigator (AC) was consulted. Eligible studies included retrospective or prospective observational studies investigating the association between variables and either 1) the risk for BCBM occurrence, or 2) the TTBM. Articles were excluded from the review if: 1) there was no reference to BM-related biomarker discovery or no statistical analysis, 2) they included patients from studies involving clinical interventions 3) they evaluated prognostic factors in mixed population cohorts (BC and other primaries), or rare BC subtypes such as inflammatory BC, or 4) they reported on prognostic factors for only distinct types of BM such as cystic BM, leptomeningeal, and intradural BM. In cases where numerous studies existed reporting on prognostic markers derived from the same patient cohort, only the latest or most comprehensive studies were included (unless different variables, or outcomes were addressed).

### Categorization of included studies

The literature review was organized based on the prognostic outcome for which variables were evaluated as outlined below:

Group A: Studies reporting prognostic factors associated with the risk for BCBM [10, 12, 15–82].

Group B: Studies reporting prognostic factors associated with the TTBM [10–12, 24, 28, 30, 32, 38, 39, 46, 47, 54–56, 58, 61, 63, 68, 70, 76, 78, 83–106].

Group C: Experimental studies investigating correlation between proteins, or gene expression profiling data with incidence of BC or TTBM [32, 34, 37, 56, 63–65, 68, 69, 72, 76, 77, 82, 85, 93, 98].

Group D: Studies reporting risk prediction models for the development of BCBM [29, 53, 71, 78, 79, 102].

### Data extraction

The data extracted from eligible studies included author details, year of publication, setting, study design, study population, year of patient recruitment, variables examined, measure of association and size, duration of follow-up, and the median TTBM. Due to discrepancies in the IHC surrogate markers of BC subtypes across studies, the joint categories of HR status and HER2 expression were noted instead. Given the large number of variables considered across studies, results focused on only the variables that were found to exhibit statistically significant association with BCBM on univariate or multivariate analysis in at least one study. Additionally, due to heterogeneity in the patient populations, treatment protocols, methodologies and reporting of results between different studies, meta-analysis was not attempted.

### Quality assessment

The Quality in Prognosis Studies (QUIPS) tool, was used to assess risk of bias in the selected studies [115]. This involves qualitative assessment of 6 key bias domains (study participation, attrition, prognostic factors measurement, outcome measurements, study confounding and statistical analysis and reporting) via the aid of 3–6 prompting questions which rank studies accordingly into low, moderate or high risk of bias [116]. In order to generate a comprehensive account of predictive and prognostic factors for BCBM, no study was excluded based on the QUIPS score.

## SUPPLEMENTARY MATERIALS







## Abbreviations

BC: Breast Cancer
BM: Brain Metastases
BCBM: Breast Cancer Brain Metastases
TTBM: Time To Brain Metastases
MRI: Magnetic Resonance Imaging
WBRT: Whole Brain Radiation Therapy
BBB: Blood Brain Barrier
IHC: Immunohistochemistry
HER2: Human Epidermal Receptor 2
TNBC: Triple-Negative Breast Cancer
HR: Hormone Receptor
ER: Estrogen Receptor
PR: Progesterone Receptor
PCI: Prophylactic Cranial Irradiation
PRISMA: Preferred Reporting Items for Systematic Reviews and Meta-Analyses
QUIPS: Quality in Prognosis Studies
IDC: Invasive Ductal Carcinoma
ILC: Invasive Lobular Carcinoma
CTCs: Circulating Tumor Cells
