# Supplementary material for: Risk factors for breast cancer brain metastases: a systematic review
Source: Oncotarget. 2020 Feb 11;11(6):650–69. doi: 10.18632/oncotarget.27453 (PMC7021234; doi:10.18632/oncotarget.27453)
Supplement: Supplementary file 1 [file oncotarget-11-650-s001.pdf]

Risk factors for breast cancer brain metastases: a systematic review

SUPPLEMENTARY MATERIALS

Supplementary Table 1: Details of the studies included in the systematic review. See Supplementary Table 1

Supplementary Table 2: QUIPS scoring

Source	1. Study Participation	2. Study Attrition	3. Prognostic factor definition and measurement	4. Outcome definition and measurement	5. Confounding measurement and handling	6. Statistical analysis and reporting
Aziz et al. 2002 [1]	High	High	Moderate	Low	Moderate	Moderate
Evans et al. 2004 [2]	Low	N/A	Moderate	Low	High	Moderate
Lai et al. 2004 [3]	Low	N/A	Moderate	Low	Low	Moderate
Gabos et al. 2006 [4]	Moderate	N/A	Low	Low	Low	Low
Hicks et al. 2006 [5]	High	N/A	Low	Low	High	Moderate
Stemmler et al. 2006 [6]	Low	High	Moderate	Low	High	Moderate
Tham et al. 2006 [7]	Low	N/A	Low	Low	Low	Low
Yau et al. 2006 [8]	Low	High	Low	Low	High	Moderate
Luck et al. 2008 [9]	Low	N/A	Low	Low	Moderate	Moderate
Duchnowska et al. 2009 [10]	Low	High	Low	Low	Low	Low
Montagna et al. 2009 [11]	Low	High	Low	Low	Low	Low
Ono et al. 2009 [12]	Low	High	Low	Low	Low	Low
Paluch-Shimon et al. 2009 [13]	Low	High	Low	Moderate	High	Moderate
Graesslin et al. 2010 [14]	Low	High	Moderate	Low	Low	Moderate
Niwinska et al. 2010 [15]	Low	Moderate	Low	Moderate	Low	Moderate
Heitz et al. 2011 [16]	Low	N/A	Low	Low	Low	Low
Mego et al. 2011 [17]	Low	High	Low	Low	Low	Low
Sanz-Pamplona et al. 2011 [18]	Moderate	N/A	Low	Low	High	Moderate
Shao et al. 2011 [19]	Moderate	N/A	Low	Low	High	High
Sihto et al. 2011 [20]	Moderate	N/A	Low	Low	High	Moderate
Dayan et al. 2012 [21]	Low	N/A	Low	Low	Moderate	Moderate
Lin et al. 2012 [22]	Low	Low	Low	Low	Low	Low
Ma et al. 2012 [23]	Low	High	Low	Low	Low	Low
Park et al. 2012 [24]	Moderate	High	Low	Moderate	Low	Low
Vaz-Luis et al. 2012 [25]	Low	High	Low	Low	Low	Moderate
Wikman et al. 2012 [26]	Moderate	N/A	Moderate	Low	High	Moderate
Demircioglu et al. 2013 [27]	Moderate	N/A	Low	Low	High	Moderate
Hess et al. 2013 [28]	Low	High	Moderate	Low	Low	Low
Ishihara et al. 2013 [29]	Low	High	Low	Moderate	Low	Low
Minisini et al. 2013 [30]	Moderate	High	Low	Low	Moderate	Moderate
Sosinska-Mielcarek et al.2013 [31]	Low	N/A	Low	Low	Low	Moderate
Xue et al. 2013 [32]	Low	Moderate	Low	Low	Low	Moderate
Hung et al. 2014 [33]	Moderate	High	Low	Low	Low	Low
Chow et al. 2015 [34]	Low	High	Low	Low	Low	Moderate
Jensen et al. 2015 [35]	Moderate	N/A	Moderate	Low	Moderate	Moderate
Martinez-Aranda et al. 2015 [36]	Moderate	High	Moderate	Low	Low	Moderate
Darlix et al. 2016 [37]	Moderate	N/A	Moderate	High	Low	Low
Li et al. 2016 [38]	High	N/A	Moderate	Moderate	Moderate	Moderate
Tonyali et al. 2016 [39]	Moderate	High	Low	Low	Low	Moderate
Wangchinda et al. 2016 [40]	Moderate	High	Low	Moderate	Low	Low
Boral et al. 2017 [41]	High	N/A	Low	Moderate	High	Moderate
Furet et al. 2017 [42]	High	N/A	Moderate	Low	Low	Moderate
Klimov et al. 2017 [43]	Moderate	High	Low	Moderate	Low	Low
Le Rhun et al. 2017 [44]	Low	Moderate	Low	Low	Low	Low
Lim et al. 2017 [45]	Moderate	High	Low	Low	Low	Low
Martin et al. 2017 [46]	Moderate	High	Low	Low	Low	Low
Martinez-Aranda et al. 2017 [47]	Moderate	N/A	Low	Low	Low	Low

Witzel et al. 2017 [48]	Moderate	N/A	Moderate	Moderate	High	Moderate
Kim et al. 2018 [49]	Low	High	Moderate	Low	High	Moderate
Maurer et al. 2018 [50]	Low	High	Low	Low	Low	Low
Eichler et al. 2008 [51]	Moderate	High	Low	Low	Low	Low
Fromm et al. 2008 [52]	Moderate	High	Moderate	Moderate	Low	Moderate
Hines et al. 2008 [53]	Low	High	Moderate	Low	High	Moderate
Melisko et al. 2008 [54]	Low	Low	Moderate	Low	Moderate	Moderate
Park et al. 2009 [55]	Low	High	Low	Low	Moderate	Moderate
Bai et al. 2010 [56]	Low	Low	Low	Low	Moderate	Low
Adamo et al. 2011 [57]	Moderate	N/A	Low	Low	High	Moderate
Arslan et al. 2011 [58],	Low	High	Low	Low	Low	Moderate
Jang et al. 2011 [59]	Low	High	Moderate	Low	High	Moderate
Duchnowska et al. 2012 [60]	Low	Moderate	Low	Low	Low	Moderate
Fokas et al. 2012 [61]	Moderate	High	Low	Low	Low	Moderate
Ulasov et al. 2012 [62]	Moderate	N/A	Low	Low	High	High
Quigley et al. 2013 [63]	Low	High	Moderate	Low	High	High
Sperduto et al. 2013 [64]	Low	Moderate	Low	Low	High	High
Tarhan et al. 2013 [65]	Low	High	Low	Low	Low	Low
Sevenich et al. 2014 [66]	High	N/A	Low	Moderate	High	Moderate
Tabouret et al. 2014 [67]	Low	Low	Low	Moderate	Moderate	Low
Bachmann et al. 2015 [68]	Moderate	High	Low	Low	High	High
Duchnowska et al. 2015 [69]	Moderate	N/A	Low	Low	Low	Low
Duchnowska et al. 2015 [70]	Moderate	High	Moderate	Low	High	Moderate
Leone et al. 2015 [71]	Moderate	Moderate	Low	Low	Low	Moderate
Grubb et al. 2016 [72]	High	Moderate	Low	Low	Low	Moderate
Kyeong et al. 2017 [73]	Moderate	Moderate	Low	Moderate	High	Moderate
Li et al. 2017 [74]	Moderate	Moderate	Low	Low	Low	Low
Darlix et al. 2018 [75]	Moderate	High	Low	Low	Low	Low
Heitz et al. 2009 [76]	Moderate	Moderate	Low	Low	Low	Moderate
Saip et al. 2009 [77]	Moderate	Moderate	Low	Low	Low	Moderate
Kwon et al. 2010 [78]	Moderate	High	Low	Low	High	High
Ray et al. 2010 [79]	Low	N/A	Low	Low	Low	Moderate
Brogi et al. 2011 [80]	Moderate	Moderate	Moderate	Low	High	Moderate
Arvold et al. 2012 [81]	Moderate	Low	Low	Low	Low	Moderate
Berghoff et al. 2012 [82]	Low	Moderate	Low	Low	Moderate	High
Dawood et al. 2012 [83]	Low	High	Low	Low	Low	Low
Xu et al. 2012 [84]	Moderate	Moderate	Low	Low	Low	Low
Yap et al. 2012 [85]	Low	Low	Low	Low	Low	Low
Zhang et al. 2013 [86]	Moderate	High	Low	Moderate	Low	Low
Aversa et al. 2014 [87]	Moderate	N/A	Low	Low	Moderate	Moderate
Berghoff et al. 2014 [88]	Moderate	N/A	Low	Moderate	Low	Low
Rudat et al. 2014 [89]	Moderate	Moderate	Moderate	Moderate	Moderate	High
Kaplan et al. 2015 [90]	Moderate	N/A	Low	Low	Low	Low
Shen et al. 2015 [91]	Moderate	High	Low	Low	Low	Low
Voduc et al. 2015 [92]	High	N/A	Low	Low	Low	Low
Zakaria et al. 2016 [93]	Moderate	N/A	Moderate	Low	Moderate	Moderate
Genre et al. 2017 [94]	Low	N/A	Low	Low	Low	Low
Qian et al. 2017 [95]	High	N/A	Moderate	Moderate	Low	Low
Azim et al. 2018 [96]	Low	High	Low	Low	Low	Low

*N/A = not applicable

Supplementary Table 3: Reported risk factors for BM in studies with unselected BC patients. See Supplementary Table 3

Supplementary Table 4: Reported risk factors for BM in HER2-positive BC patients

Prognostic factor	Comparison groups (increased vs. lower risk)	Studies measuring factor	Significant association with increased risk for BCBM on multivariate analysis	
			Univariate analysis	Multivariate analysis
**Age at primary BC diagnosis**	Case (BM+) vs Control (BM-)	**1** [6, 90]		
	<50 vs. ≥50	**3** [8, 11, 25]		**2** < 50 years, HR: 2.7, $p = 0.0048$ [11]; Young age OR: 1.66 $p = 0.014$ [25]
	≤50 vs. >50	**2** [12, 76]	**1** [12]	**1** ≤ 50 years, HR: 1.92 $P = 0.04$ [12]
	≤40 vs. >40	**1** [50]	**1** [50]	**1** ≤ 40 years, $p = 0.045$ [50]
	Continuous	**1** [10]		
	Young age	**1** [3]		**1** $p < 0.05$ [3]
**Age at MBC diagnosis (years)**	≤40 vs. >40	**1** [50]	**1** [50]	
**Hormone receptor status**	HR- vs. HR+	**7** [6, 12, 13, 25, 39, 50, 90]	**3** [6, 25, 39]	**2** Negative, OR: 1.75 $p = 0.033$ [25]; Negative, RR: 3.41 $p = 0.01$ [39]
	ER- vs. ER+	**5** [3, 10–12, 39]	**1** [39]	
	PR- vs. ER+	**3** [10, 12, 39]	**1** [39]	
**Primary tumor size (T stage)[b]**	T2–4 vs. T1	**1** [50]	**1** [50]	**1** > 2 cm, HR:4.94 $p = 0.0036$ [50]
	T3–4 vs. T1–2	**3** [12, 39, 76]	**2** [39, 76]	
**Lymph node status (N-Stage)**	Positive vs. Negative	**4** [3, 12, 50, 76]	**1** [50]	**2** NS, $p < 0.01$ [3]; N1–3, $p = 0.0045$ [50]
**Clinical stage (TNM)[c]**	I-II vs. III-IV	**1** [90]		
	III vs. I	**2** [25, 39]	**1** [39]	**2** Stage III, OR 2.05 $p = 0.020$ [25]; Stage III, RR: 9.39 $p = 0.0032$ [39]
	Stage IV	**1** [12]	**1** [12]	1 [12]
**Systemic disease control status**	Progressive disease vs. complete response, partial response/ stable disease	**1** [90]	**1** [90]	
**Number of nonbrain metastases**	≥2 vs. 1	**1** [90]	**1** [90]	**1** ≥ 2, OR: 8.30 $p < 0.001$ [90]
	≥3 vs. <3	**1** [12]	**1** [12]	
**Site of metastases**	Liver (Yes vs. No)	**4** [3, 12, 69, 90]	**2** [12, 90]	**2** $p < 0.01$ [3]; HR: 2.1, $p = 0.04$ [12]
	Lung (Yes vs. No)	**2** [3, 12]		
	Bone (Yes vs. No)	**1** [12]	**1** [12]	
	Lymph (Yes vs. No)	**1** [12]		

**First metastatic site**	Lung (not *de-novo*) vs. other than lung	**1** [50]	**1** [50]	**1** lung (not *de novo*), HR: 6.97 $p < 0.0001$ [50]
	Lung *de-novo* vs. other than lung	**1** [50]		
**Time to distant relapse**	<24 vs. ≥24 months	**3** [10, 12, 90]	**1** [10]	**1** ≤ 2 years, HR: 1.62 $p = 0.022$ [10]
	<1 year vs. ≥ 1 year	**1** [50]	**1** [50]	
**Trastuzumab use**	1 vs. ≥2	**1** [90]	**1** [90]	**1** ≥ 2 line OR: 3.43 $p = 0.003$ [90]
	No vs. Yes	**5** [3, 10, 25, 76, 85]	**1** [85]	**1** OR:1.61 $p = 0.025$ [25]
	Late (≥6 months) vs. early (<6 months) after BC diagnosis	**1** [50]		**1** Late (≥6 months after BC diagnosis), HR: 2.65, $p = 0.043$, No, HR:3.79, $p = 0.0042$ [50]
**Type of surgery**	Mastectomy vs. breast conserving surgery	**1** [50]	**1** [50]	
	No breast surgery vs. breast conserving surgery	**1** [50]		

NR – Not Reported; HR – Hazard Ratio; OR – Odds Ratio; RR – Relative risk.

REFERENCES

1. Aziz SA, Pervez S, Khan S, Kayani N, Rahbar MH. Epidermal growth factor receptor (EGFR) as a prognostic marker: an immunohistochemical study on 315 consecutive breast carcinoma patients. J Pak Med Assoc. 2002; 52:104–110. [PubMed]
2. Evans AJ, James JJ, Cornford EJ, Chan SY, Burrell HC, Pinder SE, Gutteridge E, Robertson JF, Hornbuckle J, Cheung KL. Brain metastases from breast cancer: identification of a high-risk group. Clin Oncol (R Coll Radiol). 2004; 16:345–9. https://doi.org/10.1016/j.clon.2004.03.012. [PubMed]
3. Lai R, Dang CT, Malkin MG, Abrey LE. The risk of central nervous system metastases after trastuzumab therapy in patients with breast carcinoma. Cancer. 2004; 101:810–816. https://doi.org/10.1002/cncr.20418. [PubMed]
4. Gabos Z, Sinha R, Hanson J, Chauhan N, Hugh J, Mackey JR, Abdulkarim B. Prognostic significance of human epidermal growth factor receptor positivity for the development of brain metastasis after newly diagnosed breast cancer. J Clin Oncol. 2006; 24:5658–5663. https://doi.org/10.1200/JCO.2006.07.0250. [PubMed]
5. Hicks DG, Short SM, Prescott NL, Tarr SM, Coleman KA, Yoder BJ, Crowe JP, Choueiri TK, Dawson AE, Budd GT, Tubbs RR, Casey G, Weil RJ. Breast cancers with brain metastases are more likely to be estrogen receptor negative, express the basal cytokeratin CK5/6, and overexpress HER2 or EGFR. Am J Surg Pathol. 2006; 30:1097–1104. https://doi.org/10.1097/01.pas.0000213306.05811.b9. [PubMed]
6. Stemmler HJ, Kahlert S, Siekiera W, Untch M, Heinrich B, Heinemann V. Characteristics of patients with brain metastases receiving trastuzumab for HER2 overexpressing metastatic breast cancer. Breast. 2006; 15:219–225. https://doi.org/10.1016/j.breast.2005.04.017. [PubMed]
7. Tham YL, Sexton K, Kramer R, Hilsenbeck S, Elledge R. Primary breast cancer phenotypes associated with propensity for central nervous system metastases. Cancer. 2006; 107:696–704. https://doi.org/10.1002/cncr.22041. [PubMed]
8. Yau T, Swanton C, Chua S, Sue A, Walsh G, Rostom A, Johnston SR, O'Brien ME, Smith IE. Incidence, pattern and timing of brain metastases among patients with advanced breast cancer treated with trastuzumab. Acta Oncol. 2006; 45:196–201. https://doi.org/10.1080/02841860500486630. [PubMed]
9. Luck AA, Evans AJ, Green AR, Rakha EA, Paish C, Ellis IO. The influence of basal phenotype on the metastatic pattern of breast cancer. Clin Oncol (R Coll Radiol). 2008; 20:40–45. https://doi.org/10.1016/j.clon.2007.10.002. [PubMed]
10. Duchnowska R, Dziadziuszko R, Czartoryska-Arlukowicz B, Radecka B, Szostakiewicz B, Sosinska-Mielcarek K, Karpinska A, Staroslawska E, Kubiatowski T, Szczylik C. Risk factors for brain relapse in HER2-positive metastatic breast cancer patients. Breast Cancer Res Treat. 2009; 117:297–303. https://doi.org/10.1007/s10549-008-0275-z. [PubMed]
11. Montagna E, Cancello G, D'Agostino D, Lauria R, Forestieri V, Esposito A, Silvestro L, Accurso A, De Placido S, De Laurentiis M. Central nervous system metastases in a cohort of metastatic breast cancer patients treated with trastuzumab. Cancer Chemother Pharmacol. 2009; 63:275–280. https://doi.org/10.1007/s00280-008-0737-3. [PubMed]
12. Ono M, Ando M, Yunokawa M, Nakano E, Yonemori K, Matsumoto K, Kouno T, Shimizu C, Tamura K, Katsumata N, Fujiwara Y. Brain metastases in patients who receive

trastuzumab-containing chemotherapy for HER2-overexpressing metastatic breast cancer. Int J Clin Oncol. 2009; 14:48–52. https://doi.org/10.1007/s10147-008-0797-8. [PubMed]
13. Paluch-Shimon S, Ben-Baruch N, Wolf I, Zach L, Kopolovic J, Kruglikova A, Modiano T, Yosepovich A, Catane R, Kaufman B. Hormone receptor expression is associated with a unique pattern of metastatic spread and increased survival among HER2-overexpressing breast cancer patients. Am J Clin Oncol. 2009; 32:504–508. https://doi.org/10.1097/COC.0b013e3181967d72. [PubMed]
14. Graesslin O, Abdulkarim BS, Coutant C, Huguet F, Gabos Z, Hsu L, Marpeau O, Uzan S, Pusztai L, Strom EA, Hortobagyi GN, Rouzier R, Ibrahim NK. Nomogram to Predict Subsequent Brain Metastasis in Patients With Metastatic Breast Cancer. J Clin Oncol. 2010; 28:2032–2037. https://doi.org/10.1200/JCO.2009.24.6314. [PubMed]
15. Niwinska A, Murawska M, Pogoda K. Breast cancer brain metastases: differences in survival depending on biological subtype, RPA RTOG prognostic class and systemic treatment after whole-brain radiotherapy (WBRT). Ann Oncol. 2010; 21:942–948. https://doi.org/10.1093/annonc/mdp407. [PubMed]
16. Heitz F, Rochon J, Harter P, Lueck HJ, Fisseler-Eckhoff A, Barinoff J, Traut A, Lorenz-Salehi F, du Bois A. Cerebral metastases in metastatic breast cancer: disease-specific risk factors and survival. Ann Oncol. 2011; 22:1571–1581. https://doi.org/10.1093/annonc/mdq625. [PubMed]
17. Mego M, De Giorgi U, Dawood S, Wang X, Valero V, Andreopoulou E, Handy B, Ueno NT, Reuben JM, Cristofanilli M. Characterization of metastatic breast cancer patients with nondetectable circulating tumor cells. Int J Cancer. 2011; 129:417–423. https://doi.org/10.1002/ijc.25690. [PubMed]
18. Sanz-Pamplona R, Aragues R, Driouch K, Martin B, Oliva B, Gil M, Boluda S, Fernandez PL, Martinez A, Moreno V, Acebes JJ, Lidereau R, Reyal F, et al. Expression of endoplasmic reticulum stress proteins is a candidate marker of brain metastasis in both ErbB-2+ and ErbB-2- primary breast tumors. Am J Pathol. 2011; 179:564–579. https://doi.org/10.1016/j.ajpath.2011.04.037. [PubMed]
19. Shao MM, Liu J, Vong JS, Niu Y, Germin B, Tang P, Chan AW, Lui PC, Law BK, Tan PH, Tse GM. A subset of breast cancer predisposes to brain metastasis. Med Mol Morphol. 2011; 44:15–20. https://doi.org/10.1007/s00795-010-0495-2. [PubMed]
20. Sihto H, Lundin J, Lundin M, Lehtimaki T, Ristimaki A, Holli K, Sailas L, Kataja V, Turpeenniemi-Hujanen T, Isola J, Heikkila P, Joensuu H. Breast cancer biological subtypes and protein expression predict for the preferential distant metastasis sites: a nationwide cohort study. Breast Cancer Res. 2011; 13:R87. https://doi.org/10.1186/bcr2944. [PubMed]
21. Dayan A, Koca D, Akman T, Oztop I, Ellidokuz H, Yilmaz U. The factors that have an impact on the development of brain metastasis in the patients with breast cancer. J Cancer Res Ther. 2012; 8:542–548. https://doi.org/10.4103/0973-1482.106531. [PubMed]
22. Lin NU, Vanderplas A, Hughes ME, Theriault RL, Edge SB, Wong YN, Blayney DW, Niland JC, Winer EP, Weeks JC. Clinicopathologic features, patterns of recurrence, and survival among women with triple-negative breast cancer in the National Comprehensive Cancer Network. Cancer. 2012; 118:5463–5472. https://doi.org/10.1002/cncr.27581. [PubMed]
23. Ma KK, Chau WW, Wong CH, Wong K, Fung N, Lee AJ, Choi CL, Suen DT, Kwong A. Triple negative status is a poor prognostic indicator in Chinese women with breast cancer: a ten year review. Asian Pac J Cancer Prev. 2012; 13:2109–2114. https://doi.org/10.7314/APJCP.2012.13.5.2109. [PubMed]
24. Park HS, Kim S, Kim K, Yoo H, Chae BJ, Bae JS, Song BJ, Jung SS. Pattern of distant recurrence according to the molecular subtypes in Korean women with breast cancer. World J Surg Oncol. 2012; 10:4. https://doi.org/10.1186/1477-7819-10-4. [PubMed]
25. Vaz-Luis I, Ottesen RA, Hughes ME, Marcom PK, Moy B, Rugo HS, Theriault RL, Wilson J, Niland JC, Weeks JC, Lin NU. Impact of hormone receptor status on patterns of recurrence and clinical outcomes among patients with human epidermal growth factor-2-positive breast cancer in the National Comprehensive Cancer Network: a prospective cohort study. Breast Cancer Res. 2012; 14:R129. https://doi.org/10.1186/bcr3324. [PubMed]
26. Wikman H, Sielaff-Frimpong B, Kropidlowski J, Witzel I, Milde-Langosch K, Sauter G, Westphal M, Lamszus K, Pantel K. Clinical relevance of loss of 11p15 in primary and metastatic breast cancer: association with loss of PRKCDBP expression in brain metastases. PLoS One. 2012; 7:e47537. https://doi.org/10.1371/journal.pone.0047537. [PubMed]
27. Demircioglu F, Demirci U, Akmansu M. Lymph node ratio assessment of brain metastasis in early breast cancer cases. Asian Pac J Cancer Prev. 2013; 14:1665–1667. https://doi.org/10.7314/APJCP.2013.14.3.1665. [PubMed]
28. Hess KR, Esteva FJ. Effect of HER2 status on distant recurrence in early stage breast cancer. Breast Cancer Res Treat. 2013; 137:449–455. https://doi.org/10.1007/s10549-012-2366-0. [PubMed]
29. Ishihara M, Mukai H, Nagai S, Onozawa M, Nihei K, Shimada T, Wada N. Retrospective analysis of risk factors for central nervous system metastases in operable breast cancer: effects of biologic subtype and Ki67 overexpression on survival. Oncology. 2013; 84:135–140. https://doi.org/10.1159/000345321. [PubMed]
30. Minisini AM, Moroso S, Gerratana L, Giangreco M, Iacono D, Poletto E, Guardascione M, Fontanella C, Fasola G, Puglisi F. Risk factors and survival outcomes in patients with brain metastases from breast cancer. Clin Exp Metastasis. 2013; 30:951–956. https://doi.org/10.1007/s10585-013-9594-5. [PubMed]
31. Sosinska-Mielcarek K, Duchnowska R, Winczura P, Badzio A, Majewska H, Lakomy J, Peksa R, Pieczynska B, Radecka B, Debska S, Biernat W, Jassem J. Immunohistochemical

prediction of brain metastases in patients with advanced breast cancer: the role of Rad51. Breast. 2013; 22:1178–1183. https://doi.org/10.1016/j.breast.2013.08.011. [PubMed]

32. Xue J, Peng G, Yang JS, Ding Q, Cheng J. Predictive factors of brain metastasis in patients with breast cancer. Med Oncol. 2013; 30:337. https://doi.org/10.1007/s12032-012-0337-2. [PubMed]

33. Hung MH, Liu CY, Shiau CY, Hsu CY, Tsai YF, Wang YL, Tai LC, King KL, Chao TC, Chiu JH, Su CH, Lo SS, Tzeng CH, et al. Effect of age and biological subtype on the risk and timing of brain metastasis in breast cancer patients. PLoS One. 2014; 9:e89389. https://doi.org/10.1371/journal.pone.0089389. [PubMed]

34. Chow L, Suen D, Ma KK, Kwong A. Identifying risk factors for brain metastasis in breast cancer patients: Implication for a vigorous surveillance program. Asian J Surg. 2015; 38:220–223. https://doi.org/10.1016/j.asjsur.2015.03.003. [PubMed]

35. Jensen TW, Ray T, Wang J, Li X, Naritoku WY, Han B, Bellafiore F, Bagaria SP, Qu A, Cui X, Taylor CR, Ray PS. Diagnosis of Basal-Like Breast Cancer Using a FOXC1-Based Assay. J Natl Cancer Inst. 2015; 107. https://doi.org/10.1093/jnci/djv148. [PubMed]

36. Martinez-Aranda A, Hernandez V, Guney E, Muixi L, Foj R, Baixeras N, Cuadras D, Moreno V, Urruticoechea A, Gil M, Oliva B, Moreno F, Gonzalez-Suarez E, et al. FN14 and GRP94 expression are prognostic/predictive biomarkers of brain metastasis outcome that open up new therapeutic strategies. Oncotarget. 2015; 6:44254–44273. https://doi.org/10.18632/oncotarget.5471. [PubMed]

37. Darlix A, Lamy PJ, Lopez-Crapez E, Braccini AL, Firmin N, Romieu G, Thezenas S, Jacot W. Serum NSE, MMP-9 and HER2 extracellular domain are associated with brain metastases in metastatic breast cancer patients: predictive biomarkers for brain metastases? Int J Cancer. 2016; 139:2299–2311. https://doi.org/10.1002/ijc.30290. [PubMed]

38. Li R, Zhang K, Penedo TL, Kragel CP, Grizzle WE, Hameed O, Siegal GP, Wei S. The RANK Pathway in Advanced Breast Cancer: Does Src Play a Role? Appl Immunohistochem Mol Morphol. 2016; 24:42–50. https://doi.org/10.1097/PAI.0000000000000151. [PubMed]

39. Tonyali O, Coskun U, Yuksel S, Inanc M, Bal O, Akman T, Yazilitas D, Ulas A, Kucukoner M, Aksoy A, Demirci U, Uysal M, Tanriverdi O, et al. Risk factors for brain metastasis as a first site of disease recurrence in patients with HER2 positive early stage breast cancer treated with adjuvant trastuzumab. Breast. 2016; 25:22–26. https://doi.org/10.1016/j.breast.2015.11.006. [PubMed]

40. Wangchinda P, Ithimakin S. Factors that predict recurrence later than 5 years after initial treatment in operable breast cancer. World J Surg Oncol. 2016; 14:223. https://doi.org/10.1186/s12957-016-0988-0. [PubMed]

41. Boral D, Vishnoi M, Liu HN, Yin W, Sprouse ML, Scamardo A, Hong DS, Tan TZ, Thiery JP, Chang JC, Marchetti D. Molecular characterization of breast cancer CTCs associated with brain metastasis. Nat Commun. 2017; 8:196. https://doi.org/10.1038/s41467-017-00196-1. [PubMed]

42. Furet E, El Bouchtaoui M, Feugeas JP, Miquel C, Leboeuf C, Beytout C, Bertheau P, Le Rhun E, Bonneterre J, Janin A, Bousquet G. Increased risk of brain metastases in women with breast cancer and p16 expression in metastatic lymph-nodes. Oncotarget. 2017; 8:37332–37341. https://doi.org/10.18632/oncotarget.16953. [PubMed]

43. Klimov S, Rida PC, Aleskandarany MA, Green AR, Ellis IO, Janssen EA, Rakha EA, Aneja R. Novel immunohistochemistry-based signatures to predict metastatic site of triple-negative breast cancers. Br J Cancer. 2017; 117:826–834. https://doi.org/10.1038/bjc.2017.224. [PubMed]

44. Le Rhun E, Bertrand N, Dumont A, Tresch E, Le Deley MC, Mailliez A, Preusser M, Weller M, Revillion F, Bonneterre J. Identification of single nucleotide polymorphisms of the PI3K-AKT-mTOR pathway as a risk factor of central nervous system metastasis in metastatic breast cancer. Eur J Cancer. 2017; 87:189–198. https://doi.org/10.1016/j.ejca.2017.10.006. [PubMed]

45. Lim YJ, Lee SW, Choi N, Kwon J, Eom KY, Kang E, Kim EK, Kim SW, Kim JH, Kim YJ, Kim SH, Park SY, Kim JS, et al. Failure patterns according to molecular subtype in patients with invasive breast cancer following postoperative adjuvant radiotherapy: long-term outcomes in contemporary clinical practice. Breast Cancer Res Treat. 2017; 163:555–563. https://doi.org/10.1007/s10549-017-4206-8. [PubMed]

46. Martin AM, Cagney DN, Catalano PJ, Warren LE, Bellon JR, Punglia RS, Claus EB, Lee EQ, Wen PY, Haas-Kogan DA, Alexander BM, Lin NU, Aizer AA. Brain Metastases in Newly Diagnosed Breast Cancer: A Population-Based Study. JAMA Oncol. 2017; 3:1069–1077. https://doi.org/10.1001/jamaoncol.2017.0001. [PubMed]

47. Martínez-Aranda A, Hernández V, Moreno F, Baixeras N, Cuadras D, Urruticoechea A, Gil-Gil M, Vidal N, Andreu X, Seguí MA, Ballester R, Castella E, Sierra A. Predictive and prognostic brain metastases assessment in luminal breast cancer patients: FN14 and GRP94 from diagnosis to prophylaxis. Front Oncol. 2017; 7:283. https://doi.org/10.3389/fonc.2017.00283. [PubMed]

48. Witzel I, Marx AK, Muller V, Wikman H, Matschke J, Schumacher U, Sturken C, Prehm P, Laakmann E, Schmalfeldt B, Milde-Langosch K, Oliveira-Ferrer L. Role of HYAL1 expression in primary breast cancer in the formation of brain metastases. Breast Cancer Res Treat. 2017; 162:427–438. https://doi.org/10.1007/s10549-017-4135-6. [PubMed]

49. Kim YJ, Kim JS, Kim IA. Molecular subtype predicts incidence and prognosis of brain metastasis from breast cancer in SEER database. J Cancer Res Clin Oncol. 2018; 144:1803–1816. https://doi.org/10.1007/s00432-018-2697-2. [PubMed]

50. Maurer C, Tulpin L, Moreau M, Dumitrescu C, de Azambuja E, Paesmans M, Nogaret JM, Piccart MJ, Awada A. Risk factors for the development of brain metastases in patients with HER2-positive breast cancer. ESMO Open. 2018; 3:e000440. https://doi.org/10.1136/esmoopen-2018-000440. [PubMed]

51. Eichler AF, Kuter I, Ryan P, Schapira L, Younger J, Henson JW. Survival in patients with brain metastases from breast cancer: the importance of HER-2 status. Cancer. 2008; 112:2359–2367. https://doi.org/10.1002/cncr.23468. [PubMed]

52. Fromm S, Bartsch R, Rudas M, de Vries A, Wenzel C, Steger GG, Zielinski CC, Poetter R, Dieckmann K. Factors influencing the time to development of brain metastases in breast cancer. Breast. 2008; 17:512–516. https://doi.org/10.1016/j.breast.2008.03.008. [PubMed]

53. Hines SL, Vallow LA, Tan WW, McNeil RB, Perez EA, Jain A. Clinical outcomes after a diagnosis of brain metastases in patients with estrogen- and/or human epidermal growth factor receptor 2-positive versus triple-negative breast cancer. Ann Oncol. 2008; 19:1561–1565. https://doi.org/10.1093/annonc/mdn283. [PubMed]

54. Melisko ME, Moore DH, Sneed PK, De Franco J, Rugo HS. Brain metastases in breast cancer: clinical and pathologic characteristics associated with improvements in survival. J Neurooncol. 2008; 88:359–365. https://doi.org/10.1007/s11060-008-9578-5. [PubMed]

55. Park IH, Ro J, Lee KS, Nam BH, Kwon Y, Shin KH. Trastuzumab treatment beyond brain progression in HER2-positive metastatic breast cancer. Ann Oncol. 2009; 20:56–62. https://doi.org/10.1093/annonc/mdn539. [PubMed]

56. Bai B, Yuan ZY, Liu DG, Teng XY, Wang SS. Clinical features and survival analysis of different subtypes of patients with breast cancer brain metastases. Chin J Cancer. 2010; 29:413–419. https://doi.org/10.5732/cjc.009.10643. [PubMed]

57. Adamo B, Deal AM, Burrows E, Geradts J, Hamilton E, Blackwell KL, Livasy C, Fritchie K, Prat A, Harrell JC, Ewend MG, Carey LA, Miller CR, et al. Phosphatidylinositol 3-kinase pathway activation in breast cancer brain metastases. Breast Cancer Res. 2011; 13:R125. https://doi.org/10.1186/bcr3071. [PubMed]

58. Arslan UY, Oksuzoglu B, Aksoy S, Harputluoglu H, Turker I, Ozisik Y, Dizdar O, Altundag K, Alkis N, Zengin N. Breast cancer subtypes and outcomes of central nervous system metastases. Breast. 2011; 20:562–567. https://doi.org/10.1016/j.breast.2011.07.017. [PubMed]

59. Jang G, Lee SS, Ahn JH, Jung KH, Lee H, Gong G, Kim HH, Ahn SD, Son BH, Ahn SH, Kim SB. Clinical features and course of brain metastases in triple-negative breast cancer: comparison with human epidermal growth factor receptor 2-positive and other type at single institution in Korea. Breast Cancer Res Treat. 2011; 128:171–177. https://doi.org/10.1007/s10549-011-1526-y. [PubMed]

60. Duchnowska R, Biernat W, Szostakiewicz B, Sperinde J, Piette F, Haddad M, Paquet A, Lie Y, Czartoryska-Arlukowicz B, Wysocki P, Jankowski T, Radecka B, Foszczynska-Kloda M, et al. Correlation between quantitative HER-2 protein expression and risk for brain metastases in HER-2+ advanced breast cancer patients receiving trastuzumab-containing therapy. Oncologist. 2012; 17:26–35. https://doi.org/10.1634/theoncologist.2011-0212. [PubMed]

61. Fokas E, Henzel M, Hamm K, Grund S, Engenhart-Cabillic R. Brain metastases in breast cancer: analysis of the role of HER2 status and treatment in the outcome of 94 patients. Tumori. 2012; 98:768–74. https://doi.org/10.1700/1217.13502. [PubMed]

62. Ulasov IV, Kaverina NV, Pytel P, Thaci B, Liu F, Hurst DR, Welch DR, Sattar HA, Olopade OI, Baryshnikov AY, Kadagidze ZG, Lesniak MS. Clinical significance of KISS1 protein expression for brain invasion and metastasis. Cancer. 2012; 118:2096–2105. https://doi.org/10.1002/cncr.26525. [PubMed]

63. Quigley MR, Fukui O, Chew B, Bhatia S, Karlovits S. The shifting landscape of metastatic breast cancer to the CNS. Neurosurg Rev. 2013; 36:377–382. https://doi.org/10.1007/s10143-012-0446-6. [PubMed]

64. Sperduto PW, Kased N, Roberge D, Chao ST, Shanley R, Luo X, Sneed PK, Suh J, Weil RJ, Jensen AW, Brown PD, Shih HA, Kirkpatrick J, et al. The effect of tumor subtype on the time from primary diagnosis to development of brain metastases and survival in patients with breast cancer. J Neurooncol. 2013; 112:467–472. https://doi.org/10.1007/s11060-013-1083-9. [PubMed]

65. Tarhan MO, Demir L, Somali I, Yigit S, Erten C, Alacacioglu A, Ellidokuz H, Seseogullari O, Kucukzeybek Y, Can A, Dirican A, Bayoglu V, Akyol M. The clinicopathological evaluation of the breast cancer patients with brain metastases: predictors of survival. Clin Exp Metastasis. 2013; 30:201–213. https://doi.org/10.1007/s10585-012-9528-7. [PubMed]

66. Sevenich L, Bowman RL, Mason SD, Quail DF, Rapaport F, Elie BT, Brogi E, Brastianos PK, Hahn WC, Holsinger LJ, Massague J, Leslie CS, Joyce JA. Analysis of tumour- and stroma-supplied proteolytic networks reveals a brain-metastasis-promoting role for cathepsin S. Nat Cell Biol. 2014; 16:876–888. https://doi.org/10.1038/ncb3011. [PubMed]

67. Tabouret E, Metellus P, Gonçalves A, Esterni B, Charaffe-Jauffret E, Viens P, Tallet A. Assessment of prognostic scores in brain metastases from breast cancer. Neuro-oncol. 2014; 16:421–428. https://doi.org/10.1093/neuonc/not200. [PubMed]

68. Bachmann C, Schmidt S, Staebler A, Fehm T, Fend F, Schittenhelm J, Wallwiener D, Grischke E. CNS metastases in breast cancer patients: prognostic implications of tumor subtype. Med Oncol. 2015; 32:400. https://doi.org/10.1007/s12032-014-0400-2. [PubMed]

69. Duchnowska R, Jassem J, Goswami CP, Dundar M, Gokmen-Polar Y, Li L, Woditschka S, Biernat W, Sosinska-Mielcarek K, Czartoryska-Arlukowicz B, Radecka B, Tomasevic Z, Stepniak P, et al. Predicting early brain metastases based on clinicopathological factors and gene

expression analysis in advanced HER2-positive breast cancer patients. J Neurooncol. 2015; 122:205–216. https://doi.org/10.1007/s11060-014-1704-y. [PubMed]

70. Duchnowska R, Sperinde J, Chenna A, Huang W, Weidler JM, Winslow J, Haddad M, Paquet A, Lie Y, Trojanowski T, Mandat T, Kowalczyk A, Czartoryska-Arłukowicz B, et al. Quantitative HER2 and p95HER2 levels in primary breast cancers and matched brain metastases. Neuro-oncology. 2015; 17:1241–9. https://doi.org/10.1093/neuonc/nov012. [PubMed]

71. Leone JP, Lee AV, Brufsky AM. Prognostic factors and survival of patients with brain metastasis from breast cancer who underwent craniotomy. Cancer Med. 2015; 4:989–994. https://doi.org/10.1002/cam4.439. [PubMed]

72. Grubb CS, Jani A, Wu CC, Saad S, Qureshi YH, Nanda T, Yaeh A, Rozenblat T, Sisti MB, Bruce JN, McKhann GM II, Sheth SA, Lesser J, et al. Breast cancer subtype as a predictor for outcomes and control in the setting of brain metastases treated with stereotactic radiosurgery. J Neurooncol. 2016; 127:103–110. https://doi.org/10.1007/s11060-015-2014-8. [PubMed]

73. Kyeong S, Cha YJ, Ahn SG, Suh SH, Son EJ, Ahn SJ. Subtypes of breast cancer show different spatial distributions of brain metastases. PLoS One. 2017; 12:e0188542. https://doi.org/10.1371/journal.pone.0188542. [PubMed]

74. Li R, Zhang K, Siegal GP, Wei S. Clinicopathological factors associated with survival in patients with breast cancer brain metastasis. Hum Pathol. 2017; 64:53–60. https://doi.org/10.1016/j.humpath.2017.03.022. [PubMed]

75. Darlix A, Griguolo G, Thezenas S, Kantelhardt E, Thomssen C, Dieci MV, Miglietta F, Conte P, Braccini AL, Ferrero JM, Bailleux C, Jacot W, Guarneri V. Hormone receptors status: a strong determinant of the kinetics of brain metastases occurrence compared with HER2 status in breast cancer. J Neurooncol. 2018; 138:369–382. https://doi.org/10.1007/s11060-018-2805-9. [PubMed]

76. Heitz F, Harter P, Lueck HJ, Fissler-Eckhoff A, Lorenz-Salehi F, Scheil-Bertram S, Traut A, du Bois A. Triple-negative and HER2-overexpressing breast cancers exhibit an elevated risk and an earlier occurrence of cerebral metastases. Eur J Cancer. 2009; 45:2792–2798. https://doi.org/10.1016/j.ejca.2009.06.027. [PubMed]

77. Saip P, Cicin I, Eralp Y, Karagol H, Kucucuk S, Cosar Alas R, Yavuz E, Dincer M, Saglam E, Topuz E. Identification of patients who may benefit from the prophylactic cranial radiotherapy among breast cancer patients with brain metastasis. J Neurooncol. 2009; 93:243–251. https://doi.org/10.1007/s11060-008-9769-0. [PubMed]

78. Kwon HC, Oh SY, Kim SH, Lee S, Kwon KA, Choi YJ, Cho GJ, Kim YS, Lee M, Lee JH, Kim DC, Lee HS, Cho SH, et al. Clinical outcomes and breast cancer subtypes in patients with brain metastases. Onkologie. 2010; 33:146–152. https://doi.org/10.1159/000286281. [PubMed]

79. Ray PS, Wang J, Qu Y, Sim MS, Shamonki J, Bagaria SP, Ye X, Liu B, Elashoff D, Hoon DS, Walter MA, Martens JW, Richardson AL, et al. FOXC1 is a potential prognostic biomarker with functional significance in basal-like breast cancer. Cancer Res. 2010; 70:3870–3876. https://doi.org/10.1158/0008-5472.CAN-09-4120. [PubMed]

80. Brogi E, Murphy CG, Johnson ML, Conlin AK, Hsu M, Patil S, Akram M, Nehhozina T, Jhaveri KL, Hudis CA, Seidman AD. Breast carcinoma with brain metastases: clinical analysis and immunoprofile on tissue microarrays. Ann Oncol. 2011; 22:2597–2603. https://doi.org/10.1093/annonc/mdr022. [PubMed]

81. Arvold ND, Oh KS, Niemierko A, Taghian AG, Lin NU, Abi-Raad RF, Sreedhara M, Harris JR, Alexander BM. Brain metastases after breast-conserving therapy and systemic therapy: incidence and characteristics by biologic subtype. Breast Cancer Res Treat. 2012; 136:153–160. https://doi.org/10.1007/s10549-012-2243-x. [PubMed]

82. Berghoff A, Bago-Horvath Z, De Vries C, Dubsky P, Pluschnig U, Rudas M, Rottenfusser A, Knauer M, Eiter H, Fitzal F, Dieckmann K, Mader RM, Gnant M, et al. Brain metastases free survival differs between breast cancer subtypes. Br J Cancer. 2012; 106:440–446. https://doi.org/10.1038/bjc.2011.597. [PubMed]

83. Dawood S, Lei X, Litton JK, Buchholz TA, Hortobagyi GN, Gonzalez-Angulo AM. Incidence of brain metastases as a first site of recurrence among women with triple receptor-negative breast cancer. Cancer. 2012; 118:4652–4659. https://doi.org/10.1002/cncr.27434. [PubMed]

84. Xu Z, Schlesinger D, Toulmin S, Rich T, Sheehan J. Impact of Triple-Negative Phenotype on Prognosis of Patients With Breast Cancer Brain Metastases. Int J Radiat Oncol Biol Phys. 2012; 84:612–8. https://doi.org/10.1016/j.ijrobp.2011.12.054. [PubMed]

85. Yap YS, Cornelio GH, Devi BC, Khorprasert C, Kim SB, Kim TY, Lee SC, Park YH, Sohn JH, Sutandyo N, Wong DW, Kobayashi M, Landis SH, et al. Brain metastases in Asian HER2-positive breast cancer patients: anti-HER2 treatments and their impact on survival. Br J Cancer. 2012; 107:1075–1082. https://doi.org/10.1038/bjc.2012.346. [PubMed]

86. Zhang Q, Chen J, Yu X, Ma J, Cai G, Yang Z, Cao L, Chen X, Guo X. Systemic treatment after whole-brain radiotherapy may improve survival in RPA class II/III breast cancer patients with brain metastasis. J Neurooncol. 2013; 114:181–189. https://doi.org/10.1007/s11060-013-1169-4. [PubMed]

87. Aversa C, Rossi V, Geuna E, Martinello R, Milani A, Redana S, Valabrega G, Aglietta M, Montemurro F. Metastatic breast cancer subtypes and central nervous system metastases. Breast. 2014; 23:623–628. https://doi.org/10.1016/j.breast.2014.06.009. [PubMed]

88. Berghoff AS, Bartsch R, Preusser M, Ricken G, Steger GG, Bago-Horvath Z, Rudas M, Streubel B, Dubsky P, Gnant M, Fitzal F, Zielinski CC, Birner P. Co-overexpression of HER2/HER3 is a predictor of impaired survival in breast cancer patients. Breast. 2014; 23:637–643. https://doi.org/10.1016/j.breast.2014.06.011. [PubMed]

89. Rudat V, El-Sweilmeen H, Brune-Erber I, Nour AA, Almasri N, Altuwaijri S, Fadel E. Identification of breast cancer

patients with a high risk of developing brain metastases: a single-institutional retrospective analysis. BMC Cancer. 2014; 14:289. https://doi.org/10.1186/1471-2407-14-289. [PubMed]

90. Kaplan MA, Ertugrul H, Firat U, Kucukoner M, Inal A, Urakci Z, Pekkolay Z, Isikdogan A. Brain metastases in HER2-positive metastatic breast cancer patients who received chemotherapy with or without trastuzumab. Breast Cancer. 2015; 22:503–509. https://doi.org/10.1007/s12282-013-0513-z. [PubMed]

91. Shen Q, Sahin AA, Hess KR, Suki D, Aldape KD, Sawaya R, Ibrahim NK. Breast cancer with brain metastases: clinicopathologic features, survival, and paired biomarker analysis. Oncologist. 2015; 20:466–473. https://doi.org/10.1634/theoncologist.2014-0107. [PubMed]

92. Voduc KD, Nielsen TO, Perou CM, Harrell JC, Fan C, Kennecke H, Minn AJ, Cryns VL, Cheang MCU. αB-crystallin expression in breast cancer is associated with brain metastasis. NPJ Breast Cancer. 2015; 1. https://doi.org/10.1038/npjbcancer.2015.14. [PubMed]

93. Zakaria R, Platt-Higgins A, Rathi N, Crooks D, Brodbelt A, Chavredakis E, Lawson D, Jenkinson MD, Rudland PS. Metastasis-inducing proteins are widely expressed in human brain metastases and associated with intracranial progression and radiation response. Br J Cancer. 2016; 114:1101–1108. https://doi.org/10.1038/bjc.2016.103. [PubMed]

94. Genre L, Roché H, Varela L, Kanoun D, Ouali M, Filleron T, Dalenc F. External validation of a published nomogram for prediction of brain metastasis in patients with extra-cerebral metastatic breast cancer and risk regression analysis. Eur J Cancer. 2017; 72:200–209. https://doi.org/10.1016/j.ejca.2016.10.019. [PubMed]

95. Qian J, Chen H, Ji X, Eisenberg R, Chakravarthy AB, Mayer IA, Massion PP. A 3q gene signature associated with triple negative breast cancer organ specific metastasis and response to neoadjuvant chemotherapy. Scientific reports. 2017; 7:45828. https://doi.org/10.1038/srep45828. [PubMed]

96. Azim HA, Abdel-Malek R, Kassem L. Predicting Brain Metastasis in Breast Cancer Patients: Stage Versus Biology. Clin Breast Cancer. 2018; 18:e187–e95. https://doi.org/10.1016/j.clbc.2017.08.004. [PubMed]
